# Synthesis and in vitro characterization of [^198^Au]Auranofin

**DOI:** 10.1186/s41181-025-00401-3

**Published:** 2025-11-05

**Authors:** Punita Bhardwaj, Caroline Frohner, Christopher Geppert, Christian Gorges, Winfried Brenner, Guilhem Claude, Sarah Spreckelmeyer

**Affiliations:** 1 Charité – Universitätsmedizin Berlin, Corporate Member of Freie Universität Berlin and Humboldt Universität Zu Berlin, Klinik Für Nuklearmedizin, Augustenburger Platz 1, 13353 Berlin, Germany; 2 Forschungsreaktor TRIGA Mainz, Johannes-Gutenberg-Universität Mainz, Fritz-Strassmann-Weg 2, 55128 Mainz, Germany; 3 German Cancer Consortium (DKTK), Partner Site Berlin, Berlin, Germany

**Keywords:** Auranofin, ^198^Au, Gold(i) complex, Radiotheranostics, Nuclear medicine, SPECT imaging, Redox metabolism, Cancer therapy

## Abstract

**Background:**

Radiopharmaceuticals offer targeted treatment by combining diagnostic or therapeutic radionuclides with biologically active molecules. Auranofin is the only Food and Drug Administration (FDA) approved gold(I) complex, originally developed for the treatment of rheumatoid arthritis. Recent evidence has highlighted its potential as an anticancer agent due to its ability to disrupt redox signaling, inhibit thioredoxin reductase, and impair glycolytic metabolism. This study aims to incorporate the true theranostic radionuclide ^198^Au into the Auranofin scaffold and evaluate its impact in-vitro on cancer cells.

**Results:**

Carrier-added (c.a.)^198^Au was produced via neutron activation of ^197^Au and subsequently converted into c.a. H [^198^Au] [AuCl₄]. Downscaled synthetic protocols were developed to sequentially generate c.a. [^198^Au] [Au(tht)Cl], [^198^Au] [Au(PEt₃)Cl], and [^198^Au]Auranofin. Radiochemical purity was evaluated using radio-high performance liquid chromatography, and in vitro stability was assessed in human serum albumin (HSA) over 72 h. Cytotoxic and metabolic activity were investigated in MCF7 and PC3 cancer cell lines using the cell viability assay 3-(4,5-Dimethylthiazol-2-yl)-2,5-diphenyltetrazolium bromide (MTT assay) and hexokinase assay, respectively. [^198^Au]Auranofin (c.a.) was obtained with a yield of 57.0 ± 3.2% and a radiochemical purity of 96.2 ± 3.9%. The compound demonstrated stability in human serum albumin, maintaining 96.9 ± 2.5% integrity over 72 h. In vitro studies revealed that c.a. [^198^Au]Auranofin exhibited enhanced cytotoxicity and significant hexokinase inhibition compared to its non-radioactive counterpart, while the precursor complexes remained non-toxic up to 20 µM. Viability loss was both concentration and radioactivity dependent across both cell lines.

**Conclusions:**

[^198^Au]Auranofin (c.a.) represents a stable and effective radiogold-based radiopharmaceutical agent, offering redox-targeted cytotoxicity alongside β⁻ emission mediated cell death and γ emission based imaging potential. These findings highlight c.a. [^198^Au]Auranofin as a promising radiogold-based theranostic candidate, offering dual capabilities in targeted cytotoxicity and nuclear imaging. While the in vitro results are encouraging, further in vivo and translational studies are warranted to fully evaluate its clinical potential in nuclear medicine guided cancer therapy.

**Supplementary Information:**

The online version contains supplementary material available at 10.1186/s41181-025-00401-3.

## Background

Radiopharmaceuticals are a unique class of pharmaceutical drugs in which a radioactive isotope is used for imaging or therapeutic applications. The molecule can be chemically linked to a biological vector that directs it to specific targets, typically via a linker (Dhoundiyal et al. 2024).

Diagnostic radiopharmaceuticals utilize radionuclides that emit positrons (β⁺) or gamma photons (γ), enabling non-invasive imaging of physiological and molecular processes (Ilem-Ozdemir et al. 2019). Positron Emission Tomography (PET) relies on β⁺ emitters such as ^18^F (for example (e.g.) in [^18^F]FDG for metabolic imaging (Li et al. 2022)) or ^68^Ga (e.g. in [^68^Ga]Ga-DOTATATE for neuroendocrine tumor imaging (Dotatate 2016)). Single Photon Emission Computed Tomography (SPECT) employs γ-emitting isotopes like ^99m^Tc, which is widely used in nuclear medicine imaging for a wide range of physiological mechanisms (Ahmed and Zia 2023) including myocardial perfusion ([^99m^Tc]Tc-Sestamibi) and bone scans ([^99m^Tc]Tc-Diphosphonate) due to its favorable half-life and photon energy.

Therapeutic radiopharmaceuticals incorporate beta (β⁻) or alpha (α) emitters that induce a combination of localized DNA damage, oxidative stress, mitotic arrest, and immune-mediated effects, amongst others that lead to tumor cell death (Meral Tayan and Meltem 2000; Shea et al. 2024). β⁻ emitters like ^177^Lu and ^131^I are commonly used in treating neuroendocrine tumors (e.g. [^177^Lu]Lu-DOTATATE (Mittra 2018)) and thyroid cancer (e.g. Na [^131^I]I (Silberstein et al. 2012)), respectively. Alpha emitters such as ^223^Ra and ^225^Ac deliver high linear energy transfer (LET) radiation with limited tissue penetration, making them particularly effective for micro-metastatic or hematologic malignancies (Vaidyanathan and Zalutsky 2011).

The theranostic principle combines both diagnostic and therapeutic applications in chemically analogous compounds. A prime clinical example of a clinically used theranostic pair is DOTATATE: [^68^Ga]Ga-DOTATATE (NETSPOT™) for PET imaging and its therapeutic counterpart [^177^Lu]Lu-DOTATATE (Lutathera®) used in peptide receptor radionuclide therapy (PRRT). These theranostic pairs enable consistent correlation of diagnostic imaging with subsequent therapeutic response (Bethge et al. 2004). However, studies show that these theranostic pairs, although used sequentially in treatment protocols, exhibit different biodistributions. This leads to discrepancies in tumor targeting, dosimetry, and normal organ uptake (Stenvall et al. 2022; Wong et al. 2022; Gains et al. 2011).

This motivates the development of true theranostic pairs, designed to ensure identical or highly comparable in-vivo pharmacokinetics between the diagnostic and therapeutic parts. Two principal approaches that can achieve this are by using the same element but different isotopes or using two elements in a ligand scaffold which could conveniently radiolabeled for its intended use. For example, in case of the former approach, the ^64^Cu/^67^Cu pair could be utilized, where [^64^Cu]Cu-SAR-BBN is used for PET imaging and [^67^Cu]Cu-SAR-BBN for β⁻ therapy of prostate cancer (A phase I/IIa theranostic study of 64Cu-SAR-BBN and 67Cu-SAR-BBN for identification and treatment of GRPR-expressing metastatic castrate resistant prostate cancer in patients who are ineligible for therapy with 177Lu-PSMA-617 2022; Szymański et al. 2012; Krasnovskaya et al. 2023; Kelly et al. 2020). Similarly, the iodine isotope family offers three theranostic isotopes, ^123^I (SPECT), ^124^I (PET), and ^131^I (β⁻ therapy), all usable within the same ligand scaffold for versatile imaging and treatment (e.g. as NaI for thyroid cancer treatment) (Kumar and Ghosh 2021). A radio-hybrid approach would require conjugating two radiometals in one molecule (Wood et al. 2024; Baitullina et al. 2023) or a radiometal and a radio-halogen in one molecule (Krönke et al. 2025). Ongoing research continues to optimize these strategies, particularly in the context of new metal-based radiopharmaceuticals.

In this context, the gold isotopes ^198^Au and ^199^Au offer a promising yet underexplored true theranostic pair. ^198^Au (t_1/2_ = 2.7 days) emits high-energy β⁻ particles (E_β-max_ = 0.96 MeV) and a γ-ray at 411.8 keV (95.5%), making it suitable for therapy and post therapy bio-distribution imaging (International Atomic Energy A 2021). ^198^Au (c.a.) is readily produced via a direct neutron capture reaction on natural gold (^197^Au) in a nuclear reactor, using the ^197^Au(n,γ)^198^Au reaction (Hosseini et al. 2016). ^199^Au (t₁/₂ = 3.14 days) emits lower-energy β⁻ particles (E_β-max_ = 0.452 MeV) and γ emissions at 158.4 keV (40.0%) and 208.2 keV (8.7%), ideal for diagnostic SPECT imaging (Khandaker et al. 2016). Production of non-carrier-added (n.c.a) ^199^Au typically requires the β⁻ decay of ^199^Pt, obtained from neutron irradiation of enriched ^198^Pt through the ^198^Pt(n,γ)^199^Pt → ^199^Au or using ^2^⁰⁰Hg(γ,p)^199^Au pathways (Kazakov et al. 2022). While this method allows for n.c.a ^199^Au recovery, it requires access to costly and less accessible enriched ^198^Pt and necessitates a radiochemical separation of ^199^Au from the platinum target. Another commonly attempted route involves double neutron capture on ^197^Au via the ^197^Au(n,γ)^198^Au(n,γ)^199^Au reaction. However, this method yields c.a. ^199^Au due to the natural abundance of ^197^Au and the inherently low cross-section of the second neutron capture step, resulting in low isotopic enrichment and poor overall efficiency (Tárkányi et al. 2004; Sadeghi et al. 2019; Vimalnath et al. 2016; Currie and Rohren 2025).

**Fig. 1:**
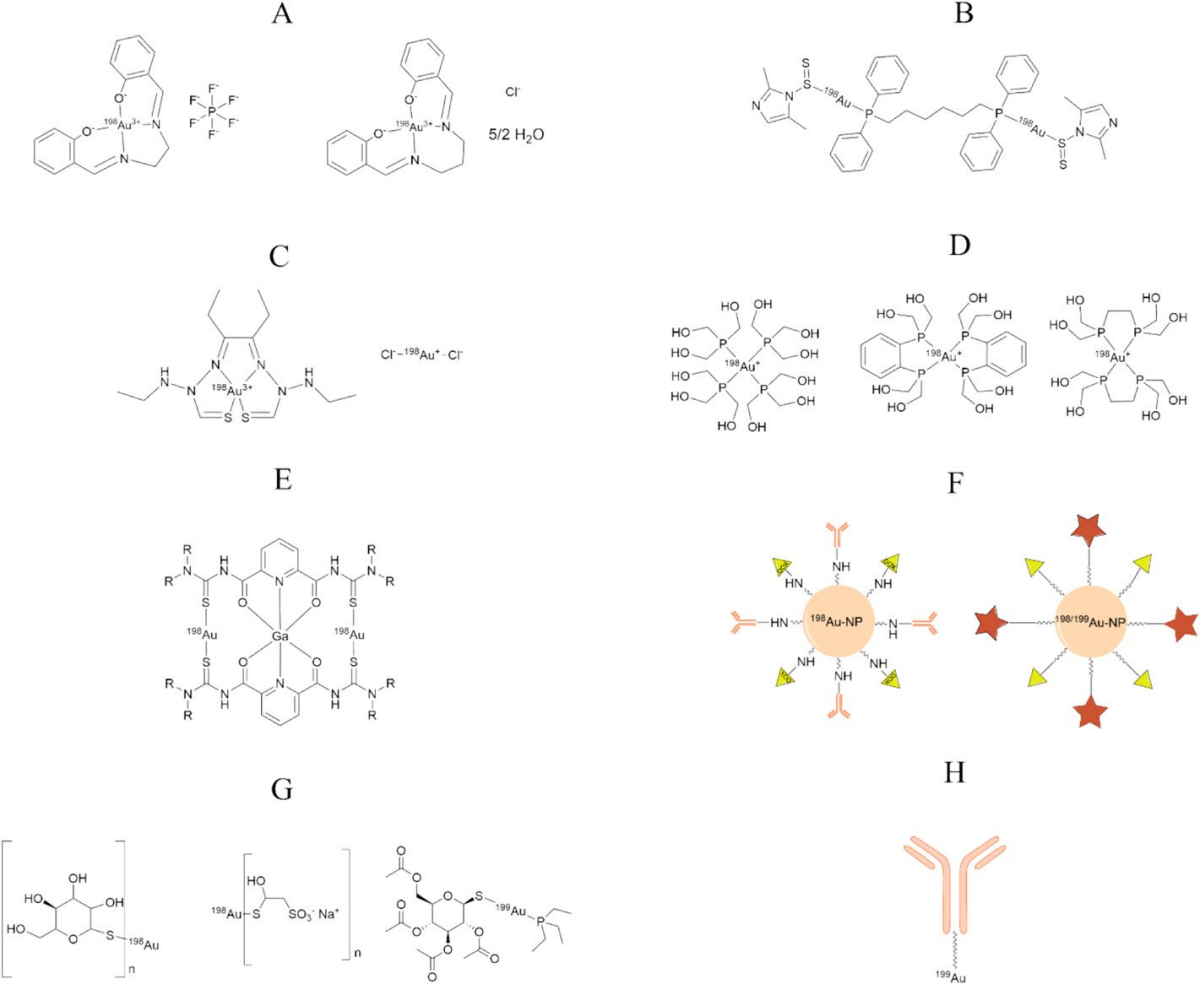
Literature derived molecular structures of radioactive gold complexes described in prior publications; **A** [^198^Au]Au–Schiff base complex (Barnholtz et al. 2001); **B** [^198^Au]Au–bisphosphinogold(I) dithiocarbamate (Kriel et al. 2015); **C** [^198^Au]Au–bis(thiosemicarbazone) complex (Bottenus et al. 2010); **D** [^198^Au]Au–hydroxymethyl phosphines (Berning et al. 1998); **E** [^198^Au]Au–metallacages (Baitullina et al. 2023); **F** Gold Nanostructures including [^198/199^Au]Au-graphene oxide nanostructures (Fazaeli et al. 2014) and [^198^Au/^199^Au]Au-Functionalized Nanoparticles (Zhao et al. 2016; Chakravarty et al. 2018; Shukla et al. 2012; Kannan et al. 2012; Davarci et al. 2023; Axiak-Bechtel et al. 2014; Chanda et al. 2010; Żelechowska-Matysiak et al. 2023; Al-Yasiri et al. 2017); **G** [^199^Au]Au–thioglucose derivatives (Atkins et al. 1975; Cottrill et al. 1989; Swartz et al. 1960); and **H** [^199^Au]Au-labeled antibodies (Garg and Hazra 2017; Anderson et al. 1988)

Only a limited number of chemically defined ^198^Au and ^199^Au radiopharmaceuticals have been explored in the literature, and most remain at the preclinical or proof-of-concept stage. Most research involving radioactive Au has focused on its incorporation into gold nano-structures (Zhao et al. 2016; Chakravarty et al. 2018; Fazaeli et al. 2014; Shukla et al. 2012; Kannan et al. 2012; Davarci et al. 2023; Axiak-Bechtel et al. 2014; Chanda et al. 2010; Żelechowska-Matysiak et al. 2023; Al-Yasiri et al. 2017) which can optionally be functionalized, while fewer approaches explore its potential in labeled small molecules. These compounds can be grouped into several key categories of small-molecule complexes such as Schiff bases (Barnholtz et al. 2001), bisphosphinogold dithiocarbamates (Kriel et al. 2015), bis(thiosemicarbazone) complexes (Bottenus et al. 2010), hydroxymethyl phosphines (Berning et al. 1998), thioglucose derivatives (Atkins et al. 1975; Cottrill et al. 1989; Swartz et al. 1960), and more elaborate platforms such as metallacages (Baitullina et al. 2023) (see Fig. 1). While these examples demonstrate diverse chemical strategies, many of them suffer from poor serum stability, non-ideal biodistribution, or lack of in vivo data.

Auranofin remains the benchmark among gold(I) complexes due to its clinical validation (Anderson et al. 1988) and biological activity (Yamashita 2021; Auranofin et al. 2007). Originally approved by the FDA for rheumatoid arthritis (Abdalbari and Telleria 2021; Roder and Thomson 2015), Auranofin is one of the few gold compounds with extensive human pharmacokinetic and safety data. Structurally, Auranofin contains a triethylphosphine ligand and a tetraacetylated glucothiolate group, making it a lipophilic drug. Once inside the body, it undergoes deacetylation in serum and ligand exchanges in cells, releasing active gold(I) species like [Au(PEt₃)]⁺ and gold-thiolate complexes (Yamashita 2021; Roder and Thomson 2015; Fiskus et al. 2014; Sabatier et al. 2021; Yang et al. 2024). These reactive species selectively bind to proteins with thiol or selenol groups, such as cysteine- or selenocysteine-containing enzymes (Albert et al. 2012; Sarra et al. 2013). Most notably, the binding to thioredoxin reductase (TrxR) impairs redox balance and mitochondrial function (Abdalbari and Telleria 2021; Radenkovic et al. 2017; Zhang et al. 2019). Detailed biochemical studies using [^35^S]S-Auranofin and [^199^Au]Auranofin showed that Auranofin enters cells by displacing the original sugar ligand through competitive exchange with membrane-bound thiol groups. Extracellular proteins such as albumin or small thiols like cysteine can also capture the gold atom. Once internalized, the gold continues thiol-mediated transfer across intracellular proteins, progressively moving from the plasma membrane to the cytoplasm. During intracellular trafficking, the phosphine ligand may be lost, further influencing redistribution. The efflux process is hypothesized to occur via the same thiol-exchange mechanism, effectively reversing the sequence of entry events (Snyder et al. 1987).

Like Cisplatin, Auranofin’s mechanism has prompted its repurposing in oncology, with multiple studies confirming cytotoxicity across diverse cancer cell lines (Sobhakumari et al. 2012; You and Park 2016; Oommen et al. 2016; Liu et al. 2014; Fhu and Ali 2021). Cancer cells exhibit elevated hexokinase expression, which supports their energy metabolism and oncogenic phenotype (Tseng et al. 2018). Auranofin has also been shown to target hexokinase and reduce its expression levels in cancer cells (An et al. 2024) and, thus, impair glycolytic metabolism by inhibiting this mitochondrial enzyme essential for glucose utilization and cell survival (Yang et al. 2024).

This study aims to first synthesize c.a. [^198^Au]Auranofin to investigate Auranofin’s intracellular behavior and therapeutic potential through its proposed dual-action mechanism pharmacologically and radiobiologically. The choice of ^198^Au over ^199^Au was guided by its high energy β⁻ emission for effective radiotherapy and its 412 keV γ emission compatible for imaging, offering dual modality utilization. Additionally, c.a. ^198^Au is more readily produced via neutron irradiation of ^197^Au, facilitating practical and scalable access for experimental studies. Secondly, the biological activity of c.a. [^198^Au]Auranofin, [^198^Au] [Au(tht)Cl], and H [^198^Au] [AuCl₄] was compared against their non-radioactive analogues in two cancer cell lines, MCF7 and PC3. For this, cell viability was quantified by the assessment of its metabolic activity through an enzymatic reduction of MTT in viable cells, as-well-as analysis of the metabolic disruption of glycolysis via quantification of the hexokinase activity. Comparing c.a. [^198^Au]Auranofin to its non-radioactive counterpart and precursor complexes disentangles the dual contributions of ligand-specific chemical cytotoxicity and β⁻ mediated radiotoxicity. The use of radioactive Auranofin allows direct evaluation of how radiogold incorporation enhances therapeutic efficacy through both targeted enzyme inhibition and radiation damage. In parallel, assessing non-radioactive and radiolabeled gold precursors enables differentiation between non-specific effects and ligand directed radio-pharmacological activity.

## Results

### Synthesis and characterization of c.a. [^198^Au]auranofin, H [^198^Au] [AuCl₄] and [.^198^Au] [Au(tht)Cl]

**Scheme 1:**
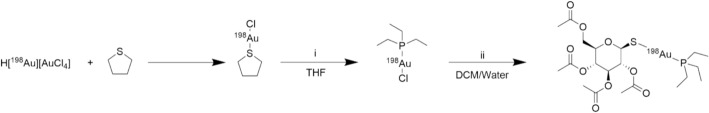
Synthetic scheme for c.a. [^198^Au]Auranofin preparation; Starting from c.a. H [^198^Au] [AuCl₄] and reacting it with tetrahydrothiohene (tht) yields [^198^Au] [Au(tht)Cl] which undergoes a reaction with triethylphosphine (PEt₃) (i) to give c.a. [^198^Au] [Au(PEt₃)Cl], and its ligand exchange with tetra-O-acetyl-1-thio-β-D-glucopyranose (ii) results in c.a. [^198^Au]Auranofin

Dissolution of 100 MBq of c.a. ^198^Au (2 mg) in freshly prepared aqua regia at 95 °C for 30 min resulted in complete solubilization, providing quantitative conversion to c.a. H [^198^Au] [AuCl₄]. Conversion to c.a. [^198^Au] [Au(tht)Cl] proceeded with a mean non decay corrected (n.d.c) reaction yield of 63.0 ± 3.7% (n = 5). Subsequent ligand exchange with triethylphosphine (PEt₃) and tetra-O-acetyl-1-thio-β-D-glucopyranose yielded c.a. [^198^Au]Auranofin with a yield of 57.0 ± 3.2% (n.d.c) (n = 5) (Scheme 1).

**Fig. 2:**
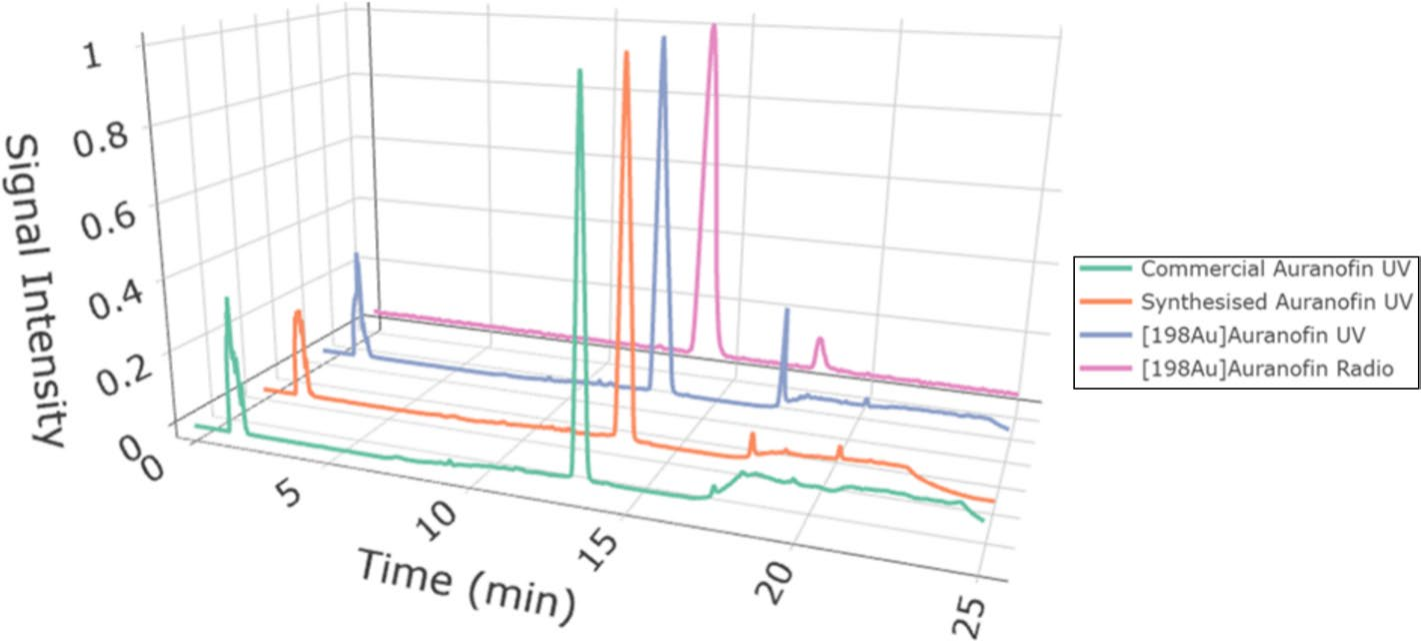
UV HPLC chromatograms of commercial Auranofin, self synthesised Auranofin and c.a. [^198^Au]Auranofin and radio-HPLC chromatogram of c.a. [^198^Au]Auranofin, in DMSO (t_R_ = 1.34 min, dead volume)

Radiochemical purity of c.a. [^198^Au]Auranofin dissolved in DMSO, as assessed by reverse-phase radio-HPLC (n = 5), was 96.2 ± 3.9% and specific activity (SA) is 0.8 MBq/mg Comparing the UV chromatograms of commercial, self-synthesized and c.a. [^198^Au]Auranofin, we observe consistent retention times of t_R(UV)_ = 13.44 min, 13.35 min and 13.21 min, respectively and t_R(Radio)_ 13.73 min. Each HPLC trace was normalized to its respective maximum intensity value. (see Fig. 2).

**Fig. 3:**
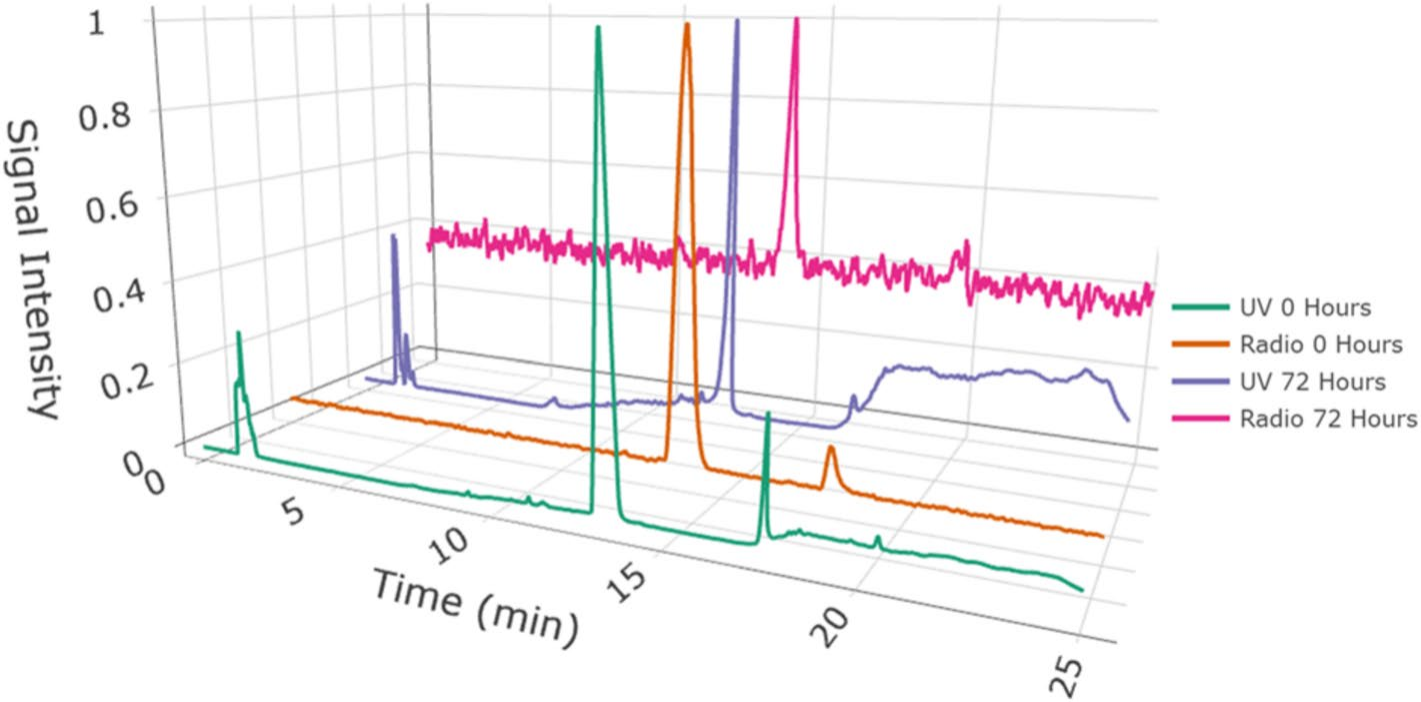
HPLC chromatograms of [^198^Au]Auranofin in DMSO (t_R_ = 1.34 min, dead volume) with UV and radio signal immediately after synthesis and after incubation in human serum albumin for 72 h

The stability of c.a. [^198^Au]Auranofin was assessed in human serum albumin at 37 °C over 72 h. [^198^Au]Auranofin (c.a.) retained a radiochemical purity of 96.9 ± 2.5% (n = 2) throughout the incubation. Quantitative analysis revealed a protein-bound fraction of 18.4 ± 6.4% and a solvent (protein unbound) fraction of 81.6 ± 6.4%, with the latter confirmed by HPLC to contain the intact radiolabeled compound. The limit of detection (LOD) of the radio-HPLC method was determined to be 0.18 kBq (10,000 c.p.s. = 1 mV) (see Fig. 3).

### Biological evaluation of cytotoxicity and hexokinase activity

The cytotoxic properties of c.a. [^198^Au]Auranofin were evaluated in human MCF7 (breast adenocarcinoma; RRID: CVCL_0031) and PC3 (prostate adenocarcinoma; RRID: CVCL_0035) cell lines using an MTT assay. Non-radioactive commercial Auranofin showed an IC₅₀ of 17.68 ± 0.10 µM for MCF7 cells and 14.33 ± 0.10 µM for PC3 cells (n ≥ 3, 6 h incubation).

To assess the additive cytotoxic effect of c.a. ^198^Au, the two cell lines were incubated with 1 µM, 10 µM and 20 µM of c.a. [^198^Au]Auranofin (SA: 0.8 MBq/mg) or non-radioactive commercial Auranofin for 6 and 72 h. The concentrations of 1, 10, and 20 µM were chosen to capture cellular effects clearly below the IC₅₀ (1 µM, “non-toxic”), just below the IC₅₀ (10 µM) and one above the IC₅₀ (20 µM “toxic”). Auranofin demonstrated dose-dependent cytotoxicity in both MCF7 and PC3 cells after 6 h and 72 h, with significant reductions in viability observed at 10 µM and 20 µM. In MCF7 cells, at 10 µM after 6 h, viability was 83.2 ± 16.3% with Auranofin and 30.3 ± 11.2% with c.a. [^198^Au]Auranofin (*p* < 0.0001). Auranofin reduced viability to 37.5 ± 9.2% at 20 µM after 6 h, while c.a. [^198^Au]Auranofin further lowered it to 21.3 ± 5.5%. At 1 µM after 72 h, c.a. [^198^Au]Auranofin decreased viability to 10.3 ± 9.6%, whereas Auranofin remained non-toxic at 115.6 ± 15.8% (p < 0.0001).

**Fig. 4:**
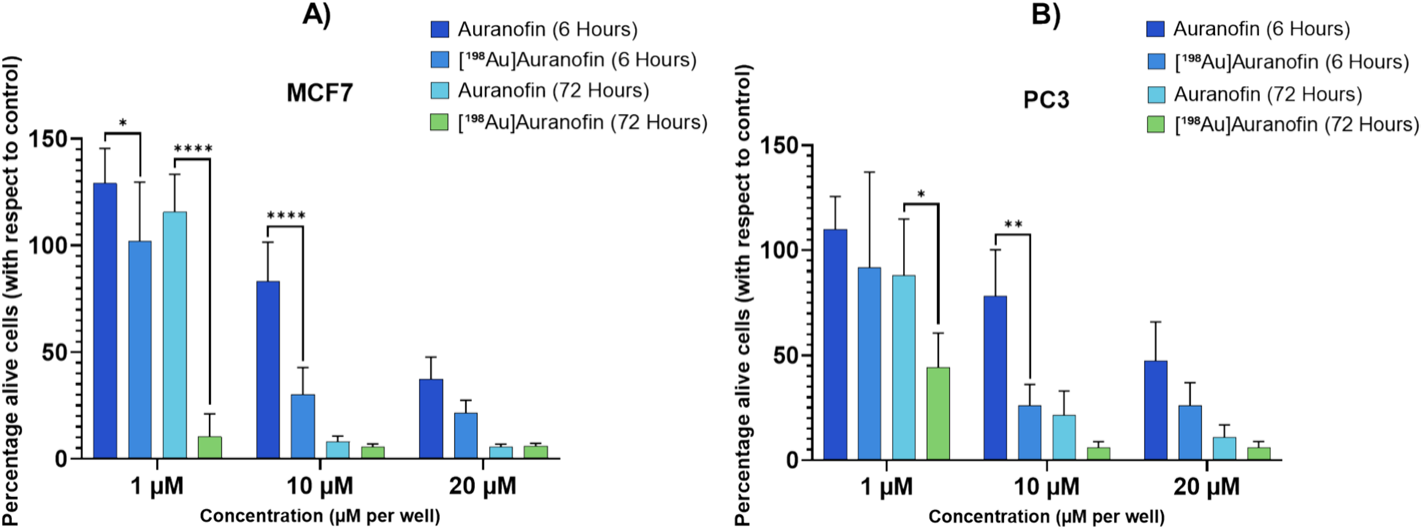
Cytotoxicity of Auranofin and c.a. [^198^Au]Auranofin (SA: 0.8 MBq/mg) showing average cell viability (± SD) relative to controls after treatment at 1, 10, and 20 µM for 6 and 72 h in **A** MCF7 and **B** PC3 cell lines. Asterisks in the figure denote levels of statistical significance: * = *P* ≤ 0.05, ** = *P* ≤ 0.01, and **** = *P* ≤ 0.0001, from Šidák’s multiple comparisons test in a one-way ANOVA analysis

Similarly, in PC3 cells, at 10 µM after 6 h, the reduction was from 78.4 ± 19.5% with Auranofin to 26.0 ± 9.0% with c.a. [^198^Au]Auranofin (p = 0.0016). Auranofin at 20 µM resulted in 47.2 ± 16.7% viability at 6 h, whereas c.a. [^198^Au]Auranofin reduced it to 26.1 ± 9.7%. At 1 µM, viability remained high after 6 h for both compounds, but after 72 h, a notable difference was observed, with Auranofin reducing viability to 88.1 ± 23.9%, while c.a. [^198^Au]Auranofin showed reduction to 44.3 ± 14.5% (p = 0.0148) (n = 5) (see Fig. 4).

To ensure that observed biological effects were specific to c.a. [^198^Au]Auranofin, we assessed the cytotoxicity of H [AuCl₄] and [Au(tht)Cl] as negative controls, along with their radiolabeled analogs c.a. H [^198^Au] [AuCl₄] (SA: 30.6 MBq/mg) and [^198^Au] [Au(tht)Cl] (SA: 2.3 MBq/mg), across a concentration range from 1 to 20 µM in both MCF7 and PC3 cells and an additional 100 µM concentration specifically for PC3 cells using MTT viability assays. No significant reduction in cell viability was observed for H [AuCl_4_] or c.a. H [^198^Au] [AuCl₄] at tested concentrations (Supplementary Figure S3.1), while a modest decrease in viability was noted specifically for [Au(tht)Cl] and c.a. [^198^Au] [Au(tht)Cl] at higher concentrations (10–20 µM) in MCF7 cells (Supplementary Figure S3.2), but this effect remained limited and did not reach levels observed for Auranofin derivatives. The comparison with [Au(tht)Cl] should be regarded as qualitative in nature, due to its inherent instability under the experimental conditions.

To evaluate the impact of c.a. [^198^Au]Auranofin on cellular metabolism, hexokinase activity was measured using a colorimetric assay in MCF7 and PC3 cells. Cells were treated with 1 µM, 10 µM and 20 µM of Auranofin and c.a. [^198^Au]Auranofin for 6 h, applying representative conditions to enable comparison with existing literature values (Sachweh et al. 2015; Freire Boullosa et al. 2021). This early time point allowed us to assess metabolic inhibition without the limitation of extensive cell death. Untreated cells served as negative controls. To distinguish whether the observed reduction in hexokinase activity was a result of direct enzymatic inhibition or simply a consequence of reduced cell viability, total cellular protein content was measured. The observed reduction in protein content at higher concentrations of Auranofin is consistent with cell death and washout during sample processing (Supplementary Figure S3.3). However, the decrease in hexokinase activity consistently exceeded the loss of protein, indicating the suppression of hexokinase activity beyond what is attributable to cell loss alone by Auranofin and c.a. [^198^Au]Auranofin. This validates specific inhibitory effect on hexokinase activity. Conversely, neither H [AuCl₄], nor [Au(tht)Cl], both radioactive and non-radioactive, exhibited significant hexokinase inhibition at any tested concentration (1 µM, 10 µM, or 20 µM) after 6 h of incubation (Supplementary Figures S3.4 & S3.5).

**Fig. 5:**
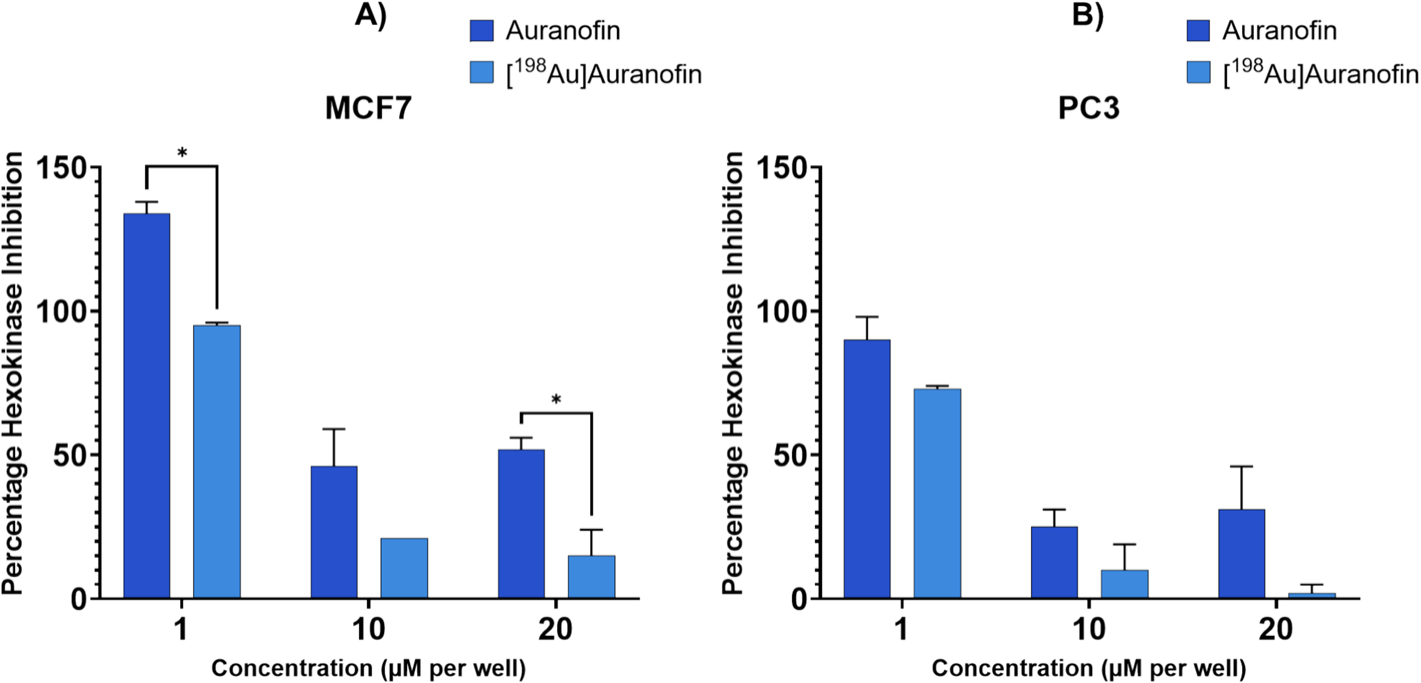
Hexokinase Inhibition of Auranofin and c.a. [^198^Au]Auranofin (SA: 0.8 MBq/mg) showing average percentage hexokinase activity (± SD) relative to controls after treatment at 1, 10, and 20 µM concentrations for 6 h in **A** MCF7 and **B** PC3 cell lines. Asterisks in the figure denote levels of statistical significance: * = *P* ≤ 0.05, from multiple t-test analysis

Hexokinase activity decreased with increasing concentrations of Auranofin and c.a. [^198^Au]Auranofin in both MCF7 and PC3 cells, aligning with concentration-dependent cytotoxicity trends seen in MTT assays. In MCF7 cells at 10 µM, Auranofin reduced hexokinase activity moderately to 46.4 ± 12.6%, while c.a. [^198^Au]Auranofin showed enhanced reduction to 20.8 ± 0.2% in MCF7 cells. At 20 µM, Auranofin reduced hexokinase activity to 52.1 ± 4.5% while c.a. [^198^Au]Auranofin led to a more pronounced suppression, with residual activity of just 15.4 ± 9.1% (p = 0.0071). Similarly in PC3 cells, 1 µM Auranofin showed a slight reduction to 89.6 ± 8.3% while c.a. [^198^Au]Auranofin further reduced it to 73.0 ± 0.8%. At 10 µM, Auranofin reduced hexokinase activity to 24.8 ± 6.5%, while c.a. [^198^Au]Auranofin showed a reduction to 10.3 ± 8.9%. At 20 µM, Auranofin showed a reduction to 30.8 ± 15.1% but c.a. [^198^Au]Auranofin showed a reduction to 1.6 ± 3.1% (n ≥ 2) (see Fig. 5).

## Discussion

The successful synthesis of c.a. [^198^Au]Auranofin from c.a. ^198^Au illustrates the feasibility of a high-purity radiolabeling scheme compatible with established organogold transformations. Despite modest yields in the conversion steps to c.a. [^198^Au] [Au(tht)Cl] (63.0 ± 3.7%) and ultimately to c.a. [^198^Au]Auranofin (57.0 ± 3.2%), the final product demonstrated high radiochemical purity of 96.2 ± 3.9%. Due to the rapid degradation of c.a. [^198^Au] [Au(tht)Cl] in solution, its radiochemical purity could not be determined (Supplementary Figure S2.6), however the synthesis route is validated using the non-radioactive analogues and confirmed via spectrometric characterization (Supplementary Figures S1.1, S1.2 and S1.3) (Bravo and Iwamoto 1969). Compared to the synthesis of [^199^Au]Auranofin performed by Cottrill et. al. (1989), which utilizes a multistep route involving [^199^Au] [Au(PEt₃)Cl] and an additional [^199^Au] [Au(PEt₃)OAc] intermediate, our approach directly converts c.a. [^198^Au] [Au(PEt₃)Cl] to c.a. [^198^Au]Auranofin. We demonstrated that the one-pot conversion of [Au(PEt₃)Cl] to Auranofin, as previously described by Wang et. al. (2017), can be successfully adapted for use with c.a. ^198^Au, yielding c.a. [^198^Au]Auranofin with high radiochemical purity and efficiency. Carrier added [^198^Au]Auranofin retains its stability under physiologically relevant conditions with 96.9 ± 2.5% integrity maintained in human serum albumin over 72 h, indicating its stability for the incubation times necessary for our in vitro experiments. The lower-than-expected protein binding contrasts with previously reported data for cold Auranofin, which shows > 90% protein binding in an excess of BSA (Schmidbaur 2009; Isab et al. 1988). This suggests that radiolabeling or assay conditions such as the buffer solution, pH, solvent and concentration of c.a. [^198^Au]Auranofin may influence binding dynamics.

Final specific activities of radiolabeled products incorporating commonly used radioisotopes like ^177^Lu and ^1^⁶^1^ Tb can directly be compared to ^198^Au, as their half-lives are similar within a clinically relevant range, making them suitable for similar therapeutic timelines. The final specific activities calculated for n.c.a. ^177^Lu and n.c.a. ^161^ Tb radiolabeled DOTATATE show high values of 4 × 10^4^ MBq/mg peptide, reflecting efficient radiolabeling and high isotope incorporation into the peptide (Lehenberger et al. 2011). This contrasts starkly with the observed 0.8 MBq/mg specific activity for [^198^Au]Auranofin, which is comparatively low, primarily due to its carrier added nature determined via the production route. This limits its effectiveness in delivering comparable radioactive payload for targeted therapy.

To establish a clear pharmacological baseline, we also investigated the gold precursors H [AuCl₄] and [Au(tht)Cl], which are widely employed in gold chemistry as Au(III) and Au(I) precursors, respectively. H [AuCl₄] is known to react with thiol and phosphine ligands (Woehrle et al. 2005), while [Au(tht)Cl] enables facile ligand exchange with donor ligands due to its labile tetrahydrothiophene ligand (Werra et al. 2023). Although chemically reactive, H [AuCl₄] has demonstrated negligible or poorly defined cytotoxic effects in cancer models, including MCF7 (Martínez-Torres et al. 2018). While [Au(tht)Cl] is well-characterized in terms of its chemical reactivity and utility in ligand exchange reactions, its biological activity remains largely unexplored. Their inclusion in our in vitro studies was therefore essential to distinguish their non-specific activity from the ligand-directed cytotoxicity of c.a. [^198^Au]Auranofin.

To disentangle the relative contributions of chemical structure and radiotoxicity, we evaluated the cytotoxic effects of Auranofin, c.a. [^198^Au]Auranofin, and their synthetic gold precursors in vitro. While Auranofin showed dose-dependent cytotoxicity, further enhanced by c.a. ^198^Au incorporation, neither H [AuCl₄] nor [Au(tht)Cl] nor their radiolabeled analogs exhibited significant toxicity at concentrations from 1 µM to 20 µM. Although c.a. [^198^Au] [Au(tht)Cl] exhibited a slight reduction in MCF7 cell viability at high concentrations (10–20 µM) after 72 h incubation, its overall cytotoxic profile remained modest and delayed compared to c.a. [^198^Au]Auranofin (Supplementary Figure S3.2). These findings reaffirm that the therapeutic potential resides in the specific ligand framework, which defines the uptake and thus the toxicity, and modulates cellular uptake and metabolic cellular mechanisms.

Carrier added [^198^Au]Auranofin displayed pronounced cytotoxicity in both MCF7 and PC3 cell lines, surpassing its non-radioactive counterpart at equivalent molar doses. This toxic enhancement was most evident at early time points highlighting the importance of tumor-specific biology in modulating its efficacy and warrants further investigation into differential intracellular processing, DNA repair dynamics, and radiobiological responses. The incorporation of c.a. ^198^Au into Auranofin significantly enhanced its cytotoxic effects compared to the non-radioactive compound. In MCF7 cells, c.a. [^198^Au]Auranofin produced an additional reduction in cell viability by 52.9 ± 21.2% at 10 µM and 16.1 ± 8.4% at 20 µM after 6 h. At 1 µM, which was otherwise non-toxic for Auranofin, [^198^Au]Auranofin induced marked delayed time-dependent cytotoxicity after 72 h, reducing cell viability to 10.3 ± 9.6% compared to the 115.6 ± 15.8% for non-radioactive Auranofin. A comparable trend was evident in PC3 cells, where c.a. [^198^Au]Auranofin led to additional reductions in viability by 52.4 ± 25.2% at 10 µM and 21.2 ± 12.7% at 20 µM after 6 h. At 1 µM, extended incubation for 72 h revealed a substantial radiotoxic effect, reducing viability by 43.7 ± 37.9% more with [^198^Au]Auranofin than Auranofin.

The effect of increasing the specific activity of [^198^Au]Auranofin correlates to enhanced radiotoxic effects, as a higher proportion of radioactive molecules per mass delivers more decay events per cell. Supplementary Figure S3.6. represents the observed effect of specific activity of [^198^Au]Auranofin on cell viability at 1 µM, 10 µM and 20 µM for 6 and 72 h. Similarly, we also analyzed the dependency on radiation dose delivered to the cells with 1 µM, 10 µM and 20 µM [^198^Au]Auranofin for 6 and 72 h incubation time points. Corresponding evidence for this dose-dependent cytotoxicity can be found in Supplementary Figure S3.7.

Although the combination of beta-radiation and the chemical toxicity of [^198^Au]Auranofin may offer synergistic advantages, several challenges must be considered in future in vivo studies. First, the beta emission from ^198^Au could damage surrounding healthy tissue if [^198^Au]Auranofin does not selectively accumulate in tumor tissue. Secondly, the systemic toxicity of non-radioactive Auranofin needs to be considered and, known side-effects from Auranofin such as diarrhea and nausea, may be exacerbated by the addition of ^198^Au.

These findings show a clear time- and dose-dependent enhancement of cytotoxicity resulting from ^198^Au decay, especially at lower concentrations where the contribution of radiotoxicity is more distinguishable. These results indicate that the β⁻ emission from ^198^Au delivers localized radiotoxicity that complements Auranofin’s redox-modulating and metabolic-inhibitory mechanisms. The differential between radioactive and non-radioactive responses narrowed by 72 h, after the decay of over half of the initial radioactivity, suggesting a saturation point where cell death is predominantly governed by chemical cytotoxicity, but impaired intracellular repair pathways due to both chemical and radiobiological damage may also play a role. Nevertheless, Carrier added [^198^Au]Auranofin exhibited a consistently greater effect across concentrations and time points.

In-vitro studies conducted with ^177^Lu directed radiopharmaceuticals demonstrate that [^177^Lu] [LuCl₃] shows a slight reduction in cell viability at concentrations up to 50 µCi across cancer lines, while targeted ^177^Lu agents yield significantly enhanced cytotoxicity at same concentrations (Jin et al. 2024; Wu et al. 2022; Rasaneh et al. 2010). Likewise, in our study, c.a. H [^198^Au] [AuCl₄] exhibited negligible cytotoxic effects, whereas c.a. [^198^Au]Auranofin produced pronounced early-phase viability losses, reinforcing the importance of ligand directed delivery for effective radiotherapeutic action.

Our findings suggest that c.a. [^198^Au]Auranofin exhibits enhanced early-phase cytotoxicity compared to its non-radioactive counterpart. Notably, the observed reduction in hexokinase activity at higher concentrations (10 and 20 µM) is likely influenced not only by direct enzyme inhibition but also by reduced cell viability. As incubation time increases to 72 h, the cytotoxic advantage diminishes, possibly due to widespread cell death masking differential effects or limiting further metabolic assessment. These data indicate that ^198^Au based radiotoxicity adds a significant metabolic burden beyond chemical inhibition alone, particularly at higher concentrations.

Taken together, these findings confirm that c.a. [^198^Au]Auranofin retains the bioactivity of its parent compound while gaining enhanced cytotoxic potential through β^⁻^ emission and the imaging capability with its γ photons. Its chemical stability, coupled with reproducible synthetic accessibility, make it a compelling scaffold for further preclinical evaluation. One limitation of this study is the reliance on 2D in vitro models, which cannot fully capture the pharmacokinetics and biodistribution dynamics in vivo. Thus, preclinical animal models in combination with radionuclide SPECT imaging will be the next step to further investigate the tumor-toxic potential of [^198^Au]Auranofin. Additionally, long-term intracellular stability and sub-organellar trafficking of c.a. [^198^Au]Auranofin warrant further investigation using subcellular fractionation or live-cell imaging with fluorescent analogs. Future work may also explore alternative gold ligands for modulating pharmacokinetics.

## Conclusion

In this study, we successfully synthesized and radiolabeled Auranofin with c.a. ^198^Au to generate c.a. [^198^Au]Auranofin, which combines the established biochemical toxic activity of Auranofin with the radiotoxic potential of β⁻ emission. The synthetic route, involving sequential formation of [Au(tht)Cl] and [Au(PEt₃)Cl] intermediates, was efficient and reproducible at small scale, yielding high-purity c.a. [^198^Au]Auranofin with excellent stability in human serum albumin over 72 h.

Biological evaluation demonstrated that c.a. [^198^Au]Auranofin exerted enhanced cytotoxicity compared to non-radioactive Auranofin in both MCF7 and PC3 cell lines, with the most significant differences observed at 6 h of incubation. Importantly, gold precursors such as H [AuCl₄] and [Au(tht)Cl] and their radioactive analogues exhibited no significant cytotoxicity up to 20 µM. Hexokinase activity assays further confirmed that c.a. [^198^Au]Auranofin enhanced the glycolytic disruption observed with Auranofin treatment.

Thus, the cytotoxic profile of c.a. [^198^Au]Auranofin aligns with its intended application as a radio-theranostic agent. The β⁻ emissions from ^198^Au contribute to additional cell death, complementing Auranofin’s inherent toxic ability to disrupt redox homeostasis. Importantly, ^198^Au also emits γ-rays suitable for SPECT imaging, allowing simultaneous monitoring of biodistribution and therapeutic binding. This dual-emission profile supports both treatment and imaging applications in a single molecular entity. Moreover, our findings suggest that ^198^Au offers a practical and effective platform for early-stage evaluation of gold-based redox-targeted radiopharmaceuticals that require both cytotoxic efficacy and traceable biodistribution.

Taken together, these findings support the further preclinical evaluation of c.a. [^198^Au]Auranofin in rodent tumor models as a candidate for true theranostics in oncology, combining effective metabolic inhibition with localized radiotoxic effects. Future studies will focus on in vivo biodistribution, therapeutic efficacy, and dosimetry to fully explore its translational potential in nuclear medicine.

## Methods

### Materials

All chemicals and solvents were of analytical or HPLC grade and used as received, including tetrahydrofuran (THF) (Thermo-Scientific, USA), acetone (Sigma-Aldrich, USA), triethylphosphine (PEt₃) (ABCR, Germany), and 1-thio-β-D-glucose tetraacetate (Sigma-Aldrich, USA). Chloro(tetrahydrothiophene)gold(I) [Au(tht)Cl] was synthesized from hydrogen tetrachloroaurate(III) H [AuCl₄] and tetrahydrothiophene (Sigma-Aldrich, USA) according to established procedures (Uson et al. 1989).

Carrier added radiogold precursor ^198^Au(0) was produced by neutron activation of high-purity (99.9%) metallic gold (Degussa, Germany) at the TRIGA research reactor (Mainz, Germany). Radioactivity was quantified using an ISOMED 2010 dose calibrator (Nuvia Instruments, Germany). TraceSelect water (Sigma-Aldrich, USA) was used in radiolabeling protocols.

Radio-HPLC analyses were conducted using a JASCO PU-980 system equipped with a JASCO UV-975 UV detector (JASCO, Japan) and Ramona Star radio-detector with a BGO crystal coincidence cell (Raytest GmbH, Germany). Data acquisition was performed using GINA X chromatography software (Raytest GmbH, Germany). Chromatographic separation employed an RSC Gel C18ec, 125 × 4.0 mm, 5 μm column. Mobile phases comprised H₂O (solvent A) and acetonitrile (solvent B), both containing 0.1% trifluoroacetic acid (Sigma-Aldrich, USA). Gradient elution was as follows: 0–4 min 10% B, 4–8 min 10–60% B, 8–15 min 60% B, 15–16 min 60–95% B, 16–22 min 95% B, 22–23 min 95–10% B, 23–25 min 10% B. HPLC retention times were verified with commercial Auranofin standards (TargetMol, USA), and radiochemical purity (> 90%) was confirmed prior to biological assays.

Disposable plastics for cell culture (Falcon; Corning; Thermo Scientific, USA and Sarstedt, Germany) were sterile, pyrogen-free, and handled under aseptic conditions in a Class II standard biosafety cabinet (KOJAIR, Finland). All radiochemical manipulations were performed in compliance with institutional radiation safety regulations.

### Physical characterization

Nuclear magnetic resonance (^1^H and ^31^P NMR) spectra were acquired on a JEOL 400 MHz spectrometer (JEOL, Japan). Electrospray ionization time-of-flight mass spectrometry (ESI-TOF MS) was performed with Agilent 6210 (Agilent Technologies, USA) under the following conditions: flow rate of 4 μL/min, spray voltage of 3.8 kV, and desolvation gas pressure maintained at 15 psi. Selected NMR, and MS spectra supporting compound characterization are included in the Supporting Information (Supplementary Figure S1.1, S1.2 and S1.3).

### Synthesis of tetrachloroauric acid H [AuCl₄]

Elemental gold (50 mg, 252.57 µmol) was dissolved in freshly prepared aqua regia (300 µL, concentrated HCl: concentrated HNO₃ = 3:1) by heating at 99 °C until complete dissolution was observed. The acidic solution was then evaporated to dryness at 99 °C to remove excess solvents. The residue was reconstituted in 200 µL of 0.1 M hydrochloric acid to afford a stock solution of H [AuCl₄], which was subsequently used as the precursor for further synthesis steps.

### Synthesis of chloro(tetrahydrothiophene)gold(I) [Au(tht)Cl]

[Au(tht)Cl] was synthesized following a downscaled method adapted from the protocol described by Uson et. al. (1989). To a solution of H [AuCl₄] in 0.1 M HCl (200 uL, 86.07 mg, 252.57 µmol) tetrahydrothiophene (100 µL, 1.13 mmol, 4.5 eq.) was added. Upon addition, a transient yellow–orange precipitate formed immediately, which gradually converted to a fine white solid upon shaking with a vortex mixer. To this ethanol (300 µL, 80% v/v) was added and vortexed before centrifugation at 700 rpm for 90 s. The supernatant was carefully decanted and the residue washed with diethyl ether (500 µL). This washing step was done twice to remove all traces of water which was found to have a deleterious effect on the subsequent reaction(s). The resulting solid was air dried under an air stream to yield [Au(tht)Cl] as a white powder suitable for further use. Yield – 68% (55.23 mg, 171.7 µmol).

### Synthesis of chloro(triethylphosphine)gold(I) [Au(PEt₃)Cl]

Chloro(triethylphosphine)gold(I) [Au(PEt₃)Cl] was prepared by ligand exchange following a modified downscaled procedure adapted from Vihervaara et. al. (2023). To a suspension of [Au(tht)Cl] (55.23 mg, 171.75 µmol) in tetrahydrofuran (500 µL), triethylphosphine (185 µL of a 20% v/v solution in ethanol, 257.62 µmol, 1.5 eq.) was added. The reaction mixture was vortexed vigorously for 1 min at room temperature. It changed from a suspension to a clear solution. The solvent was evaporated under a gentle stream of air to afford [Au(PEt₃)Cl] as an oily residue, to which diethyl ether was added and dried under gentle air stream to yield [Au(PEt₃)Cl] as a precipitate. Yield – 85% (51.1 mg, 145.7 µmol).

### Synthesis of (2,3,4,6-Tetra-O-acetyl-1-thio-β-D-glucopyranosato)(triethylphosphine)gold(I) Auranofin

(2,3,4,6-Tetra-O-acetyl-1-thio-β-D-glucopyranosato)(triethylphosphine)gold(I), Auranofin was synthesised using a procedure modified from Wang et. al. (2017). The crude [Au(PEt₃)Cl] was dissolved in dichloromethane (140 µL) yielding a yellow solution. To this mixture, tetra-O-acetyl-1-thio-β-D-glucopyranose (80 mg, 219.56 µmol) in dichloromethane (160 µL) was added. Potassium carbonate (35 mg, 253.24 µmol,) in Milli-Q water (300 µL) was then added to form a biphasic mixture which was vortexed for 3 min at room temperature until the DCM phase turns clear. The aqueous layer was removed via a micropipette, and the organic layer containing Auranofin was dried under a gentle air stream, after which the residual solvents were removed by addition of diethyl ether and subsequent drying of the solid. The resulting product was dissolved in dimethylsulfoxide and used directly for further studies without additional purification. Yield – 98.27% (97.14 mg, 143.17 µmol).

^1^H NMR (400 MHz, CDCl3, ppm) (50 mg scale): δ 5.16 – 5.02 (m, 3 H, H-1, H-3, H-4), 4.94 (mc, 1 H, H-2), 4.21 (dd, J = 4.8, 12.4 Hz, 1 H, H-6a), 4.07 (dd, J = 2.4, 12.2 Hz, 1 H, H-6b), 3.70 (m, 1 H, H-5), 2.04 (s, 3 H, C(O)CH_3_), 2.02(s, 3 H, C(O)CH_3_), 1.98(s, 3 H, C(O)CH_3_), 1.95 (s, 3 H, C(O)CH_3_), 1.82 (mc, 6 H, CH_2_CH_3_), 1.19 (mc, 9 H, CH_2_CH_3_);

^31^P NMR (400 MHz, CDCl_3_): δ 37.2

ESI^+^ MS (m/z): [Auranofin + Na]^+^ 701.1238 (calculated 701.13); [(Auranofin)_2_ + Na]^+^ 1379.2568 (calculated 1379.26).

### Production of c.a. ^198^Au

Carrier-added ^198^Au was produced by thermal neutron activation of 2.3 mg of high-purity (99.9%) metallic gold via the ^197^Au(n,γ)^198^Au nuclear reaction. Irradiation was performed at the TRIGA Mainz research reactor, operating at 100 kW thermal power with a neutron flux of approximately 4.2 × 10^12^ neutrons/cm^2^/s for 247 min, yielding 100 MBq of c.a. ^198^Au gold nugget.

### Synthesis of c.a. H [^198^Au] [AuCl₄]

The c.a. ^198^Au nugget (2.3 mg, 100 MBq) was dissolved in aqua regia at 95 °C, evaporated to dryness, and reconstituted in 0.1 M HCl (230 µL) to yield a stock solution of c.a. H [^198^Au] [AuCl₄]. All yields for c.a. ^198^Au-compounds further synthesized were based on non-decay corrected radiochemical yields.

### Synthesis of c.a. [^198^Au] [Au(tht)Cl]

To c.a. H [^198^Au] [AuCl₄] (20 µL, 1 µmol, 5.703 MBq) non-radioactive H [^197^Au] [AuCl₄] (80 µL, 4 µmol) in 0.1 M HCl was added to afford a carrier added mixture of c.a. H [^198^Au] [AuCl₄] (100 µL, 5.0 µmol). To this, tetrahydrothiophene (2 µL, 22.7 µmol, 4.5 eq.) was added and vigorously shaken. This precipitate was washed with 80% ethanol and diethyl ether and subsequently centrifuged at 700 rpm for 90 s before being dried under an air stream to yield c.a. [^198^Au] [Au(tht)Cl]. Yield—69% (3.96 MBq).

### Synthesis of c.a. [^198^Au] [Au(PEt₃)Cl]

To c.a. [^198^Au] [Au(tht)Cl] (3.96 MBq) in tetrahydrofuran (200 µL), triethylphosphine (3.7 µL of a 20% v/v in ethanol, 5.08 µmol, 1.5 eq.) was added and vortexed for 1 min after which the solvent was evaporated under an air stream. The obtained residue was directly used without further purification. Yield—96% (3.79 MBq).

### Synthesis of c.a. [^198^Au]auranofin

Carrier-added [Au(PEt₃)Cl] (3.79 MBq) dissolved in dichloromethane (140µL) and tetra-O-acetyl-1-thio-β-D-glucopyranose (1.60 mg, 4.39 µmol, 1.3 eq.) in dichloromethane (160 µL) were mixed along with Potassium Carbonate (0.7 mg, 5.06 µmol, 1.5 eq.) in Milli-Q water (300 µL). The biphasic mixture was vortexed for 1 min and the aqueous layer was discarded. The organic layer containing c.a. [^198^Au]Auranofin was dried with an air stream and dissolved in dimethylsulfoxide for further studies. Yield—90% (3.41 MBq).

### Human serum albumin (HSA) stability assay

The in-vitro stability of c.a. [^198^Au]Auranofin in human serum albumin was evaluated at 37 °C. HSA (Sigma-Aldrich, USA) was mixed with radiolabeled c.a. [^198^Au]Auranofin in DMSO in a 1:1 ratio and incubated at 37 °C with gentle agitation. At designated time points, 50 µL aliquots were removed and treated with 50 µL ice-cold ethanol followed by 50 µL ice-cold acetonitrile to precipitate proteins. Samples were centrifuged (14,000 rpm, 10 min, 0 °C), and the supernatant was collected. The radioactivity in the supernatant fraction was measured using a ISOMED 2010 dose calibrator (Nuvia Instruments, Germany). An aliquot (20 µL) of the supernatant was analyzed by radio-HPLC.

### Cell culture conditions

MCF7 (human breast adenocarcinoma) and PC3 (human prostate adenocarcinoma) cell lines were maintained at 37 °C in a humidified atmosphere containing 5% CO₂. Cells were cultured in RPMI-1640 medium (Corning, USA) supplemented with 10% heat-inactivated fetal bovine serum (Gibco, USA) and 1% Penicillin–Streptomycin (Gibco, USA). Sub culturing was performed using 0.25% Trypsin–EDTA (Sigma-Aldrich, USA) when cultures reached approximately 75–80% confluency. Cells were used at passages 5–30 for all experiments to ensure reproducibility and minimize phenotypic drift. Prior to viability assays, cells were evaluated for morphology and confluence. All procedures were conducted under aseptic conditions in a certified standard biosafety cabinet.

### Cell viability assay (MTT assay)

Cell viability was quantified using the MTT colorimetric assay (Invitrogen, USA), following established protocols. MCF7 and PC3 cells were seeded in 96-well flat-bottom tissue culture plates (Falcon, USA) at a density of 20,000 cells (6 h) and 10,000 cells (72 h) per well in 200 µL of complete growth medium. Plates were incubated for 24 h at 37 °C in a humidified atmosphere with 5% CO₂ to allow for cell attachment and stabilization. Cells were treated with the radioactive (1 µM, 10 µM, and 20 µM) and non-radioactive (0.1 µM, 0.5 µM, 1 µM, 5 µM, 7.5 µM, 10 µM, 20 µM, and 25 µM) H [AuCl₄], [Au(tht)Cl] and Auranofin. Additionally, 50 µM, 75 µM and 100 µM concentrations were tested for H [AuCl₄] and [Au(tht)Cl]. The compounds were supplemented in media, while control wells received media only. Treatments were applied for 6 and 72 h, depending on the specific experimental conditions. Following the treatment period, the incubation media was aspirated and the wells were washed with sterile PBS before 200 µL of freshly prepared MTT solution (0.5 mg/mL in sterile PBS) was added to each well, and plates were incubated for an additional 4 h at 37 °C. Formed Formazan crystals were solubilized by adding 200 µL of dimethyl sulfoxide (DMSO, ≥ 99.9%) per well, followed by gentle agitation on an orbital shaker for 1 min at room temperature to ensure complete dissolution. Absorbance was recorded at 570 nm using a microplate spectrophotometer (SpectraMax Plus 384, Molecular Devices, USA). Viability was calculated relative to untreated control wells, and each treatment condition was performed in a minimum of triplicates to ensure statistical robustness. IC₅₀ values were calculated from dose–response curves using GraphPad Prism 10 (GraphPad Software, USA) and are reported as mean ± SD from at least three independent experiments.

### Hexokinase activity assay

Hexokinase enzymatic activity was assessed using the Hexokinase Assay Kit (Abcam, UK), following the manufacturer’s protocol with minor modifications to suit adherent cell-based measurements. MCF7 and PC3 cells were seeded in 96-well plates (Falcon, USA) at a density of 20,000 cells per well in 200 µL of complete growth medium and incubated for 24 h at 37 °C in a humidified 5% CO₂ atmosphere to allow for proper attachment and recovery. After 24 h, the wells were aspirated and the cells were treated with H [AuCl₄], [Au(tht)Cl] and Auranofin at 0.01 µM, 0.1 µM, 0.5 µM, 1 µM, 5 µM, 10 µM, and 20 µM concentrations but c.a. H [^198^Au] [AuCl₄], [^198^Au] [Au(tht)Cl] and [^198^Au]Auranofin were tested only at 1 µM, 10 µM, and 20 µM concentrations (0.34, 0.74 and 1.74 MBq/mg) in supplemented medium for 6 h. Post-treatment, the medium was aspirated, and cells were washed once with ice-cold PBS. All subsequent steps were conducted on ice unless stated otherwise to preserve enzyme integrity. Cells were lysed directly in-well by the addition of 70 µL of the lysis buffer provided in the kit, followed by gentle pipetting to ensure complete lysis. From the lysate, 15 µL (MCF7) or 10 µL (PC3) was transferred to a fresh well for the assay and reaction mixture as prepared by the manufacturer’s protocol was added before measuring the absorbance at 450 nm using a microplate spectrophotometer (SpectraMax Plus 384, Molecular Devices, USA), and kinetic readings were recorded every 5 min over a 110-min period to monitor real-time enzymatic conversion. Hexokinase activity was compared to untreated control samples and expressed as relative percentage. Graphs were plotted and reported as mean ± SD and an unpaired Student’s t-test was conducted using GraphPad Prism 10 (GraphPad Software, USA) (n ≥ 2) on the data set.

### BCA assay

To evaluate total protein content, we employed the Pierce™ BCA Protein Assay Kit (Thermo Scientific, Cat. No. 23225), following the manufacturer's microplate protocol. After treatment of cells (20,000 cells per well in a 96-well plate), wells were washed with ice cold PBS and aspirated followed by lysis with 70 µL lysis buffer from the Hexokinase activity assay kit. 25 µL of the resulting cell supernatant was transferred to a new plate. Each sample was then incubated with 200 µL of the BCA working reagent. The plate was incubated at 37 °C for 30 min, after which absorbance was measured at 562 nm using a microplate reader. Protein concentrations were calculated using a standard curve generated with serial dilutions of bovine serum albumin (BSA) standards in lysis buffer from the Hexokinase activity assay kit. Graphs were plotted using GraphPad Prism 10 (GraphPad Software, USA) and reported as mean ± SD.

## Electronic Supplementary Material

Below is the link to the electronic supplementary material.

Supplementary Material

## Data Availability

The datasets generated and/or analyzed during the current study are available from the corresponding author upon reasonable request.

## Abbreviations

Au(tht)Cl: Chloro(tetrahydrothiophene)gold(I)
c.a.: Carrier added
c.p.s.: Counts per second
E_βmax_: Maximum beta emission energy
e.g.: For example
ESI-TOF MS: Electrospray ionization time-of-flight mass spectrometry
FDA: Food and drug administration
H [AuCl₄]: Tetrachloroauric acid (gold(III) chloride)
HPLC: High performance liquid chromatography
HSA: Human serum albumin
IC₅₀: Half maximal inhibitory concentration
kBq: Kilobecquerel
MBq: Megabecquerel
MCF7: Michigan cancer foundation-7 (human breast adenocarcinoma cell line)
min(s): Minute(s)
MS: Mass Spectrometry
MTT: 3-(4,5-Dimethylthiazol-2-yl)-2,5-diphenyltetrazolium bromide
mV: Millivolts
n: Number of replicates in experiment
n.c.a.: Non-carrier added
n.d.c.: Non-decay corrected
NMR: Nuclear magnetic resonance
PC3: Prostate cancer-3 (human prostate adenocarcinoma cell line)
PET: Positron emission tomography
PRRT: Peptide receptor radionuclide therapy
RSC: Research scientific column
RRID: Research resource identifier
SPECT: Single photon emission computed tomography
t_1/2_: Half-life
tht: Tetrahydrothiophene
THF: Tetrahydrofuran
TrxR: Thioredoxin reductase
µM: Micromolar
UV: Ultraviolet
