# Supplementary material for: Synthesis and in vitro characterization of [198Au]Auranofin
Source: EJNMMI Radiopharm Chem. 2025 Nov 5;10:71. doi: 10.1186/s41181-025-00401-3 (PMC12589756; doi:10.1186/s41181-025-00401-3)
Supplement: Supplementary file 1 — Supplementary Material [file 41181_2025_401_MOESM1_ESM.docx]

Supplementary Information

**Gold in Motion: Synthesis and In Vitro Characterization of [¹⁹⁸Au]Auranofin for Theranostic Cancer Strategies**

**Authors Block**

Punita Bhardwaj^1^, Caroline Frohner^1^, Christopher Geppert^2^, Christian Gorges^2^, Winfried Brenner^1,3^, Guilhem Claude^1±^, Sarah Spreckelmeyer^1*±^

1. Charité – Universitätsmedizin Berlin, corporate member of Freie Universität Berlin and Humboldt Universität zu Berlin, Klinik für Nuklearmedizin, Augustenburger Platz 1, 13353 Berlin, Germany
2. Forschungsreaktor TRIGA Mainz, Johannes-Gutenberg-Universität Mainz, Fritz-Strassmann-Weg 2, 55128 Mainz, Germany
3. German Cancer Consortium (DKTK), partner site Berlin, Berlin, Germany

*corresponding author: Sarah Spreckelmeyer, [sarah.spreckelmeyer@charite.de](mailto:sarah.spreckelmeyer@charite.de), Charité - Universitätsmedizin Berlin, corporate member of Freie Universität Berlin, Humboldt-Universität zu Berlin, and Berlin Institute of Health, Department of Nuclear Medicine, Augustenburger Platz 1, 13353, Berlin, Germany

^±^ shared last-author

***Table Of Contents***

Inhalt

[*Section 1: Chemical Characterization* 3](#_Toc203760579)

[Figure S1.1.^1^H Nuclear Magnetic Resonance (NMR) spectrum of Auranofin in CDCl_3_ (δ, ppm) 3](#_Toc203760577)

[Figure S1.2.^31^P Nuclear Magnetic Resonance (NMR) spectrum of Auranofin in CDCl_3_ (δ, ppm) 3](#_Toc203760578)

[Figure S1.3. Electron Spray Ionization Mass Spectrum (ESI^+^ MS) of Auranofin 4](#_Toc202470938)

[*Section 2: High Performance Liquid Chromatographic Characterization* 5](#_Toc203760579)

[Figure S2.1. HPLC chromatogram (isocratic – 60% MeCN/40% H_2_O) of Auranofin in DMSO starting from A) 1 mg B) 0.5 mg and C) 0.1 mg gold 5](#_Toc203760581)

[Figure S2.2. HPLC chromatogram (isocratic – 60% MeCN/40% H_2_O) of reaction products produced with ligand substitution from [Au(PEt₃)Cl] to Auranofin conducted in A) DCM/H_2_O and B) THF 6](#_Toc203760582)

[Figure S2.3. HPLC chromatogram (isocratic – 60% MeCN/40% H_2_O) of reaction products produced with solvent evaporation done at 60°C for A) 5 minutes and B) 60 minutes 6](#_Toc203760583)

[Figure S2.4. HPLC chromatogram (isocratic – 60% MeCN/40% H_2_O) of reaction products produced with solvent evaporation done for 5 minutes at A) 60°C B) 99°C and C) Air Drying 7](#_Toc203760584)

[Figure S2.5. HPLC chromatogram of Auranofin with A) Protocol 1 and B) Protocol 2 8](#_Toc203760585)

[Figure S2.6. HPLC chromatogram of [Au(tht)Cl] immediately after dissolution in DMSO 8](#_Toc203760586)

[*Section 3: In-Vitro Studies* 9](#_Toc203760587)

[Figure S3.1. MTT assay following treatment for 6 and 72 hours with H[AuCl_4_] and H[^198^Au][AuCl₄], of left, MCF7 (1-20 µM, n=2) and right, PC3 (1-100 µM, n=3) cells. Data represent percentage average cell viability (± SD) relative to controls 9](#_Toc203760588)

[Figure S3.2. MTT assay following treatment for 6 and 72 hours with [Au(tht)Cl] and [^198^Au][Au(tht)Cl], of left, MCF7 (1-20 µM, n=2) and right, PC3 (1-100 µM, n=3) cells. Data represent percentage average cell viability (± SD) relative to controls 9](#_Toc203760588)

[Figure S3.3. BCA assay following treatment for 6 hours with 1 µM, 10 µM and 20 µM of radioactive and non-radioactive Auranofin, H[AuCl_4_] and [Au(tht)Cl], of MCF7 (left) and PC3 (right) cells. Data represent percentage average cell protein in µg/mL (± SD) relative to controls (n = 2) 1](#_Toc203760590)0

[Figure S3.4. Hexokinase assay following treatment for 6 hours with 1 µM, 10 µM and 20 µM of H[AuCl4] (n = 4) and H[^198^Au][AuCl4] (n = 2) of MCF7 (left) and PC3 (right) cells. Data represent percentage average hexokinase activity (± SD) relative to controls 1](#_Toc203760590)0

[Figure S3.5. Hexokinase assay following treatment for 6 hours with 1 µM, 10 µM and 20 µM of [Au(tht)Cl] (n = 4) and [^198^Au][Au(tht)Cl] (n = 2) of MCF7 (left) and PC3 (right) cells. Data represent percentage average hexokinase activity (± SD) relative to controls 1](#_Toc203760590)0

[Figure S3.6. MTT assay following treatment for 6 and 72 hours with 1 µM, 10 µM and 20 µM of [¹⁹⁸Au]Auranofin plotted against the corresponding specific activity (MBq/mg) for A) MCF7 and B) PC3 cells. Data represent percentage average cell viability relative to controls 1](#_Toc203760590)1

Figure S3.7. MTT assay following treatment for an incubation time period of A) 6 hours and B) 72 hours with 1 µM, 10 µM and 20 µM of [¹⁹⁸Au]Auranofin plotted against the corresponding dose received (mGy). Data represent percentage cell viability relative to controls. 11

Section 1: Chemical Characterization


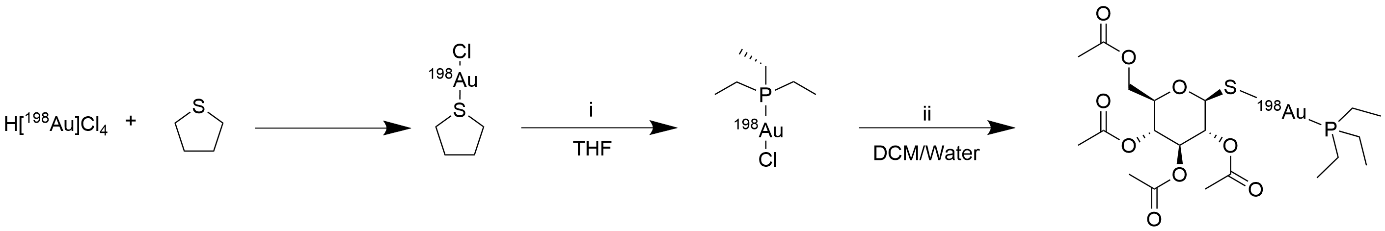


## Figure S1.1.^1^H Nuclear Magnetic Resonance (NMR) spectrum of Auranofin in CDCl_3_ (δ, ppm)


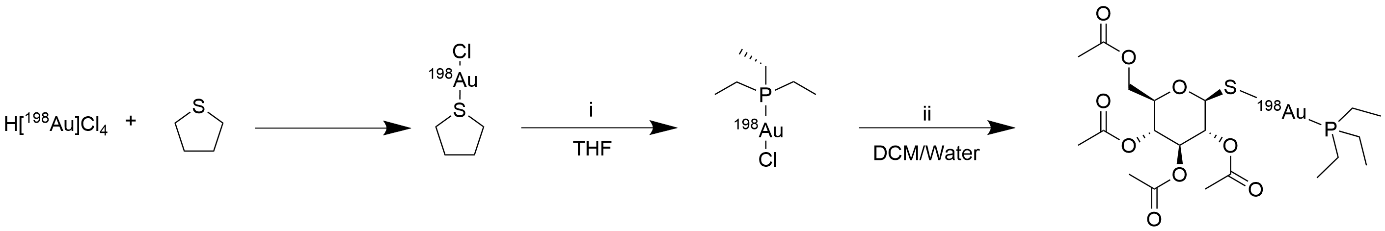


## Figure S1.2.^31^P Nuclear Magnetic Resonance (NMR) spectrum of Auranofin in CDCl_3_ (δ, ppm)


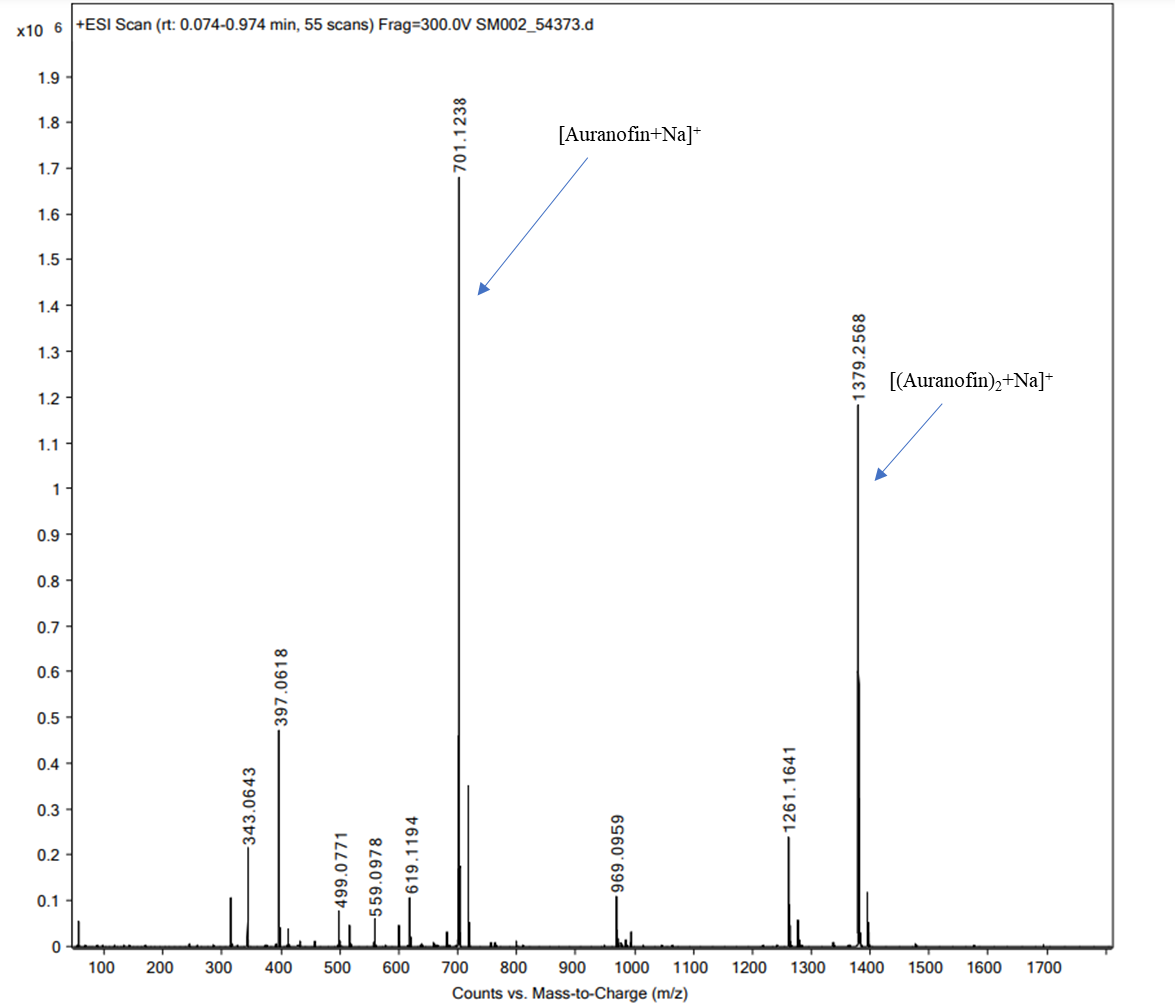


#### Figure S1.3. Electron Spray Ionization Mass Spectrum (ESI^+^ MS) of Auranofin (m/z): [Auranofin+Na]^+^ - 701.1238 (calculated. 701.13); [(Auranofin)_2_+Na]^+^ - 1379.2568 (calculated. 1379.26).

# Section 2: High Performance Liquid Chromatographic Characterization

| **Step** | **Condition** | **Observation** | **Figure** |
| --- | --- | --- | --- |
| Scale | - 1. 1 mg   2. 0.5 mg   3. 0.1 mg | Successful synthesis at all scales | Fig. S2.1. |
| Solvent | 1. 2^nd^ reaction in DCM/H_2_O 2. All reactions in THF | No product observed in THF | Fig. S2.2. |
| Heating duration | 1. Heating for 5 min at 60°C 2. Heating for 60 min at 60°C | Degradation with prolonged heating | Fig. S2.3. |
| Heating temperature | 1. Heating at 60°C 2. Heating at 99°C 3. Air drying | Air drying preserved integrity | Fig. S2.4. |


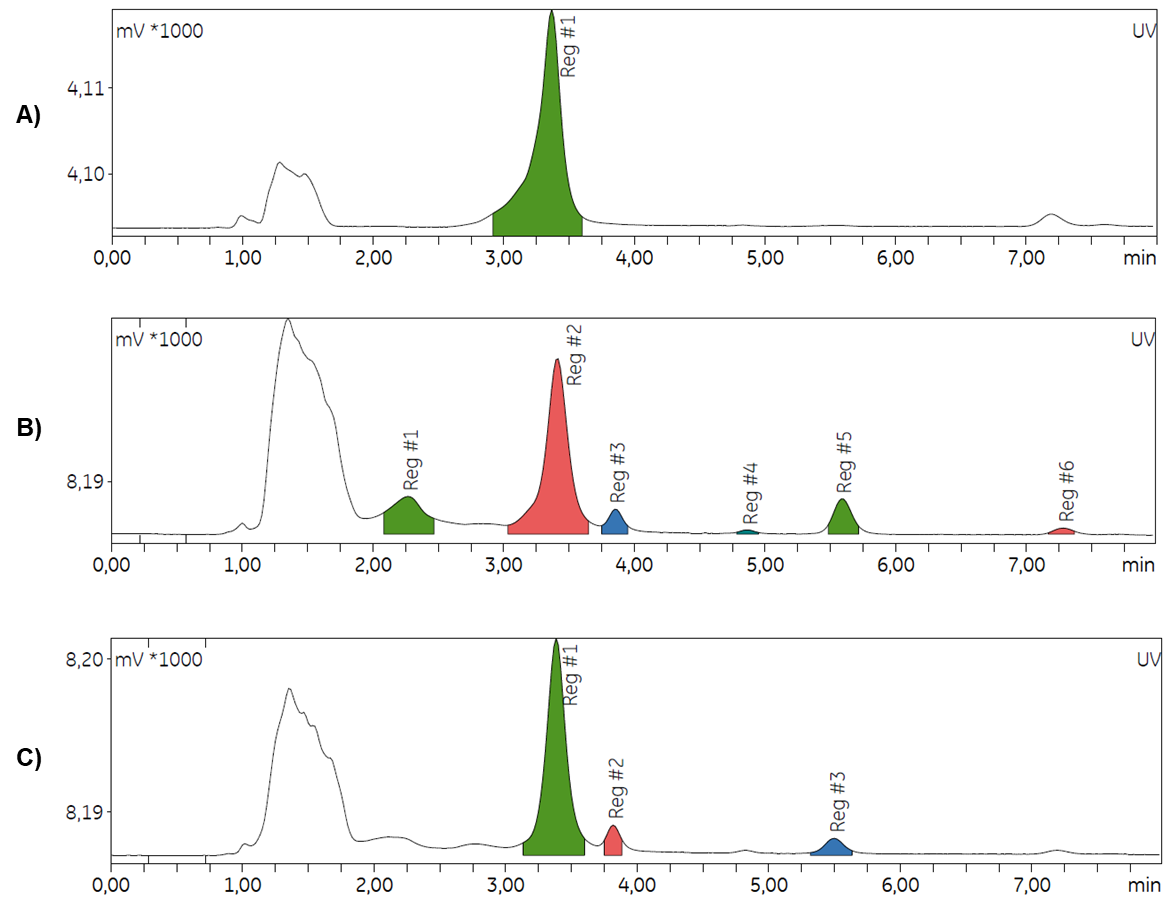


## Figure S2.1. HPLC chromatogram (isocratic – 60% MeCN/40% H_2_O) of Auranofin in DMSO starting from A) 1 mg B) 0.5 mg and C) 0.1 mg gold


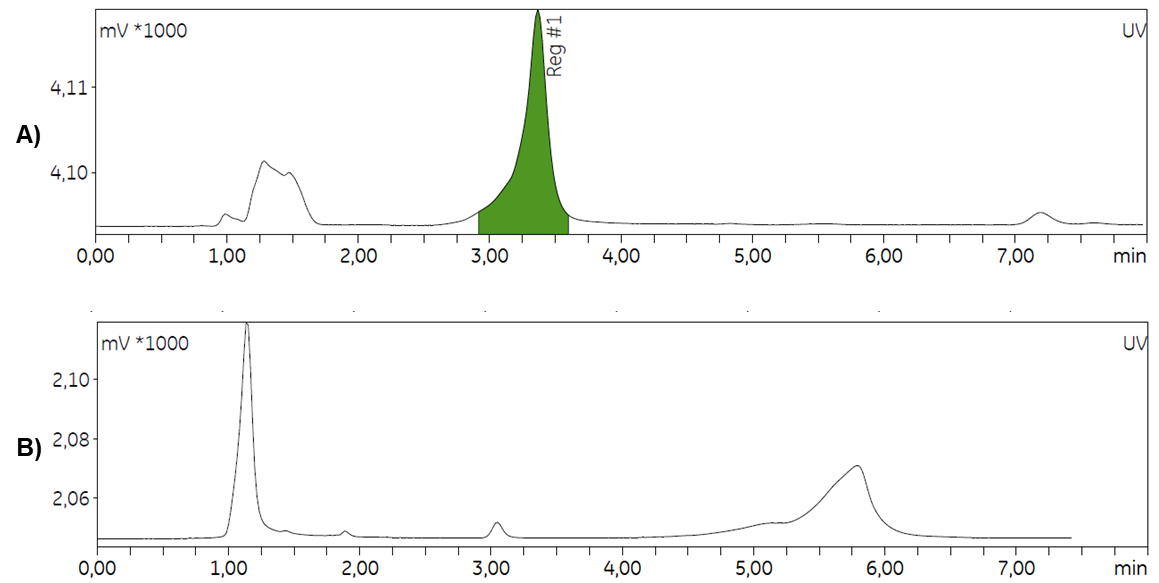


## Figure S2.2. HPLC chromatogram (isocratic – 60% MeCN/40% H_2_O) of reaction products produced with ligand substitution from [Au(PEt₃)Cl] to Auranofin conducted in A) DCM/H_2_O and B) THF


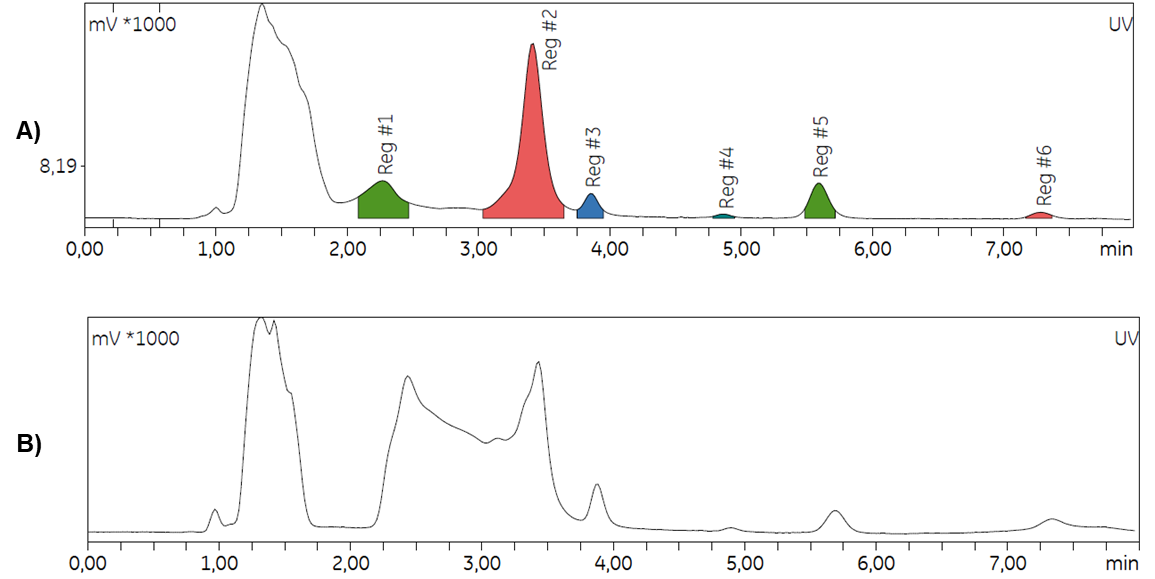


## Figure S2.3. HPLC chromatogram (isocratic – 60% MeCN/40% H_2_O) of reaction products produced with solvent evaporation done at 60°C for A) 5 minutes and B) 60 minutes


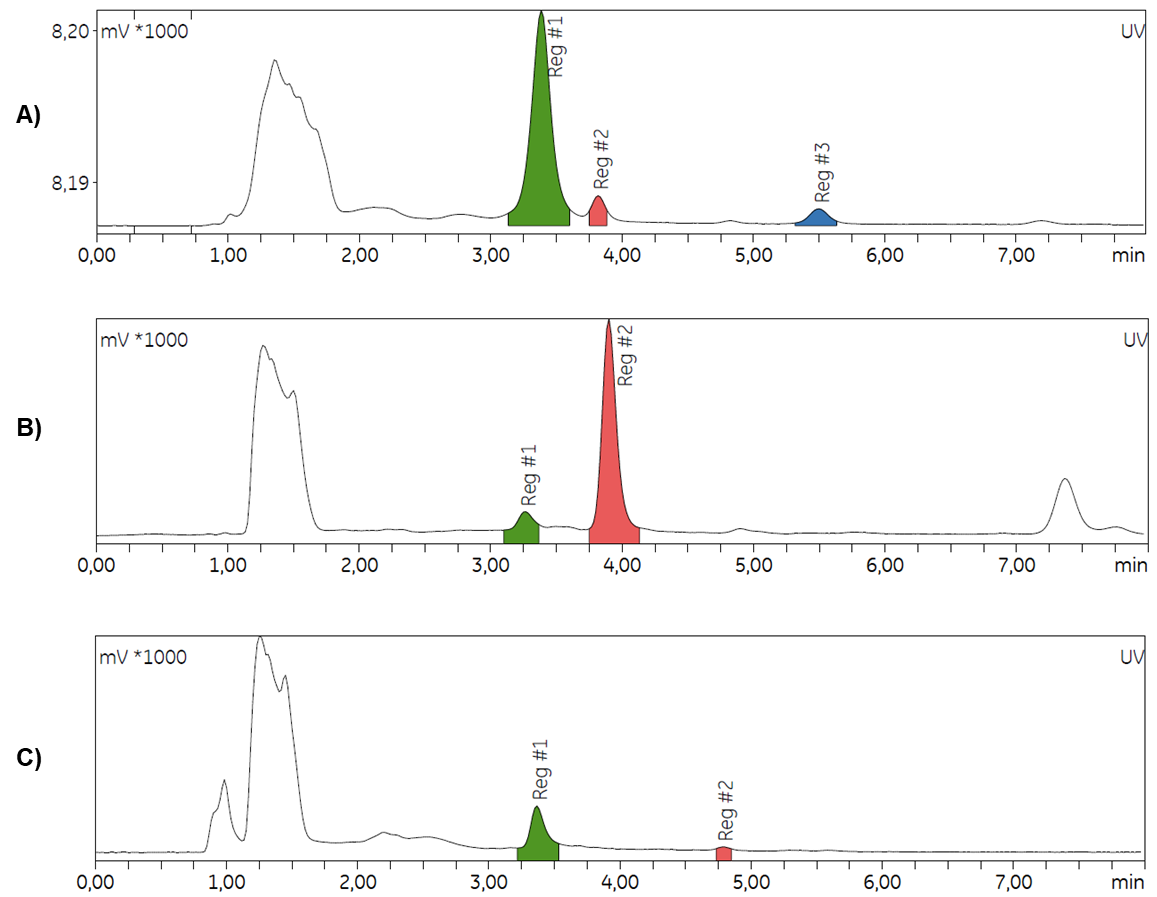


## Figure S2.4. HPLC chromatogram (isocratic – 60% MeCN/40% H_2_O) of reaction products produced with solvent evaporation done for 5 minutes at A) 60°C B) 99°C and C) Air Drying

HPLC analysis protocols were optimized for better and sharper peak shape. The protocols are listed following,

Program 1 - Isocratic elution using 60% MeCN 40% H_2_O

Program 2 - 0–4 min 10% B, 4–8 min 10–60% B, 8–15 min 60% B, 15–16 min 60–95% B, 16–22 min 95% B, 22–23 min 95–10% B, 23–25 min 10% B, where H₂O with 0.1% TFA is solvent A and acetonitrile with 0.1% TFA is solvent B.


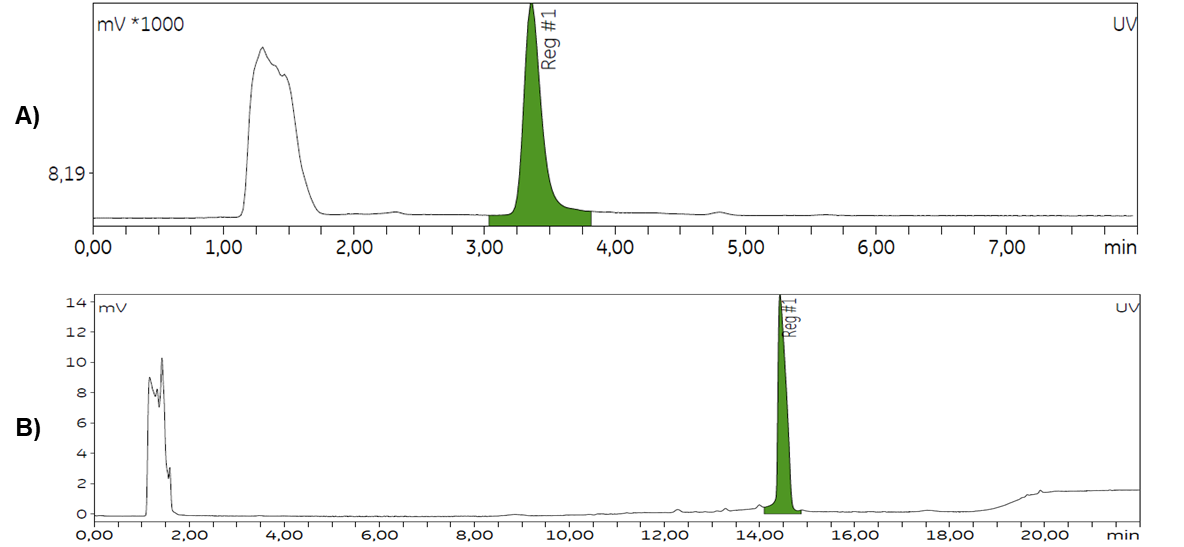


## Figure S2.5. HPLC chromatogram of Auranofin with A) Protocol 1 and B) Protocol 2

[Au(tht)Cl] was found to be unstable under the applied analytical conditions, displaying rapid degradation during analysis.


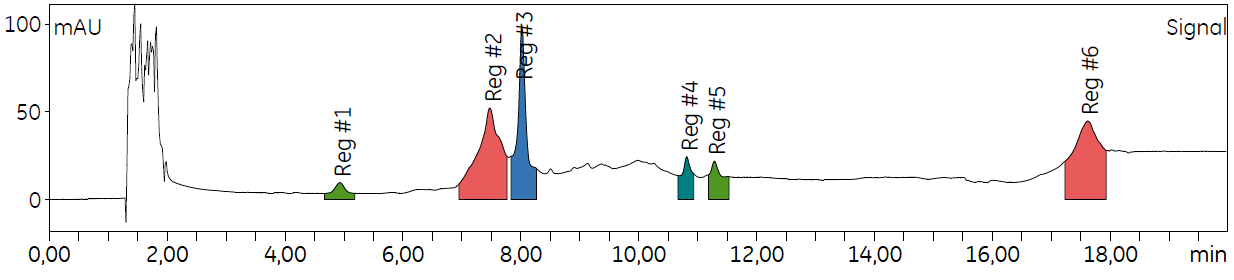


## Figure S2.6. HPLC chromatogram of [Au(tht)Cl] immediately after dissolution in DMSO

# Section 3: In-Vitro Studies


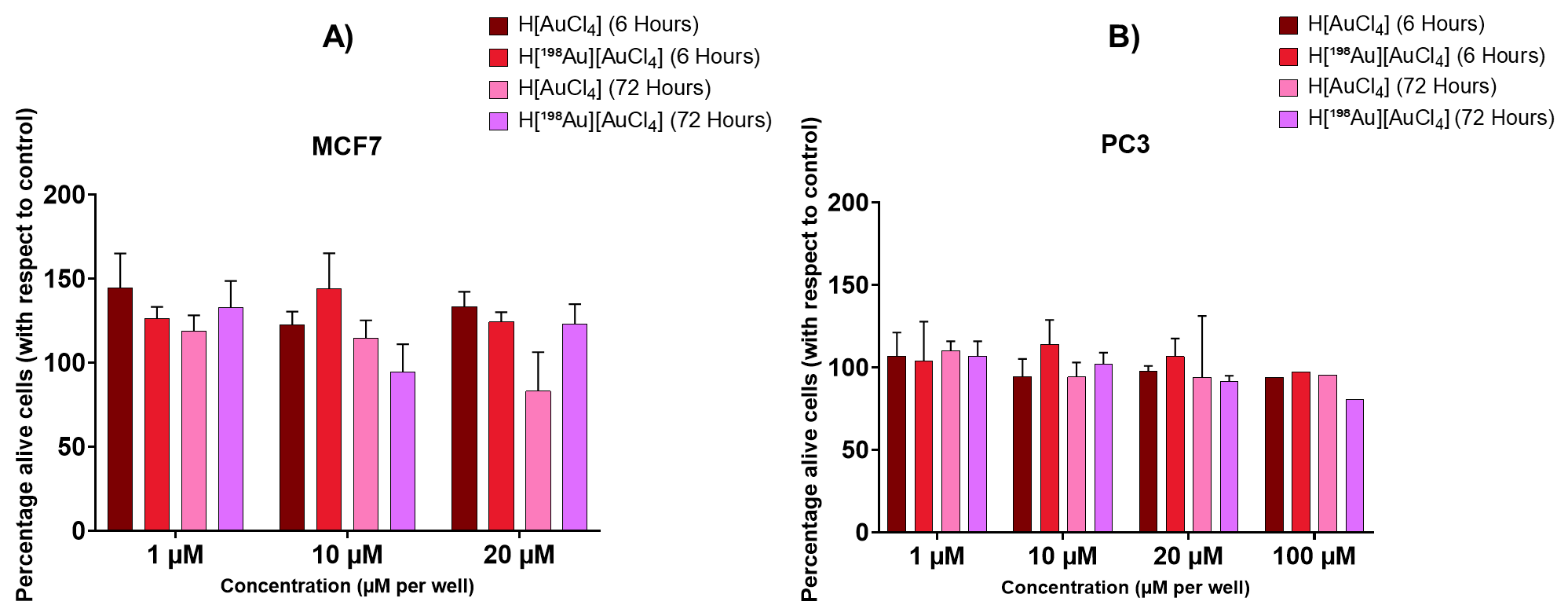


## Figure S3.1. MTT assay following treatment for 6 and 72 hours with H[AuCl_4_] and H[^198^Au][AuCl₄], of A) MCF7 (1-20 µM, n=2) and B) PC3 (1-100 µM, n = 3) cells. Data represent percentage average cell viability (± SD) relative to controls


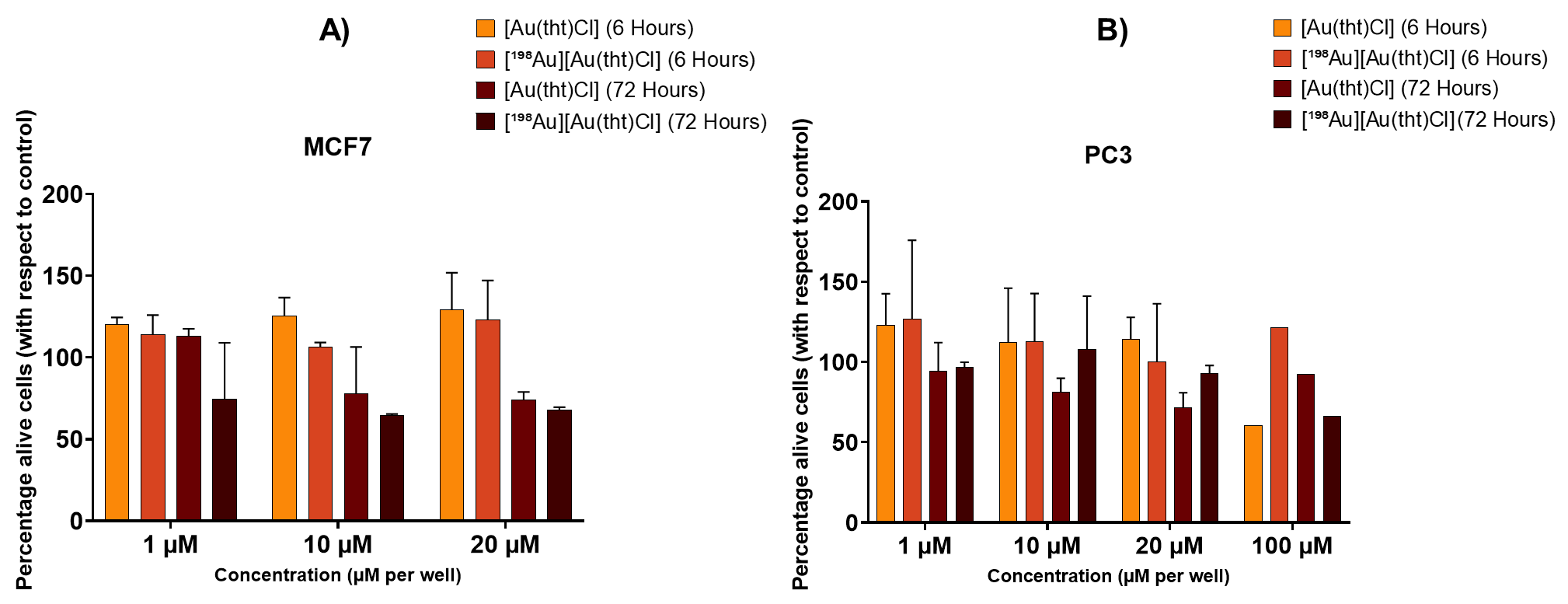


Figure S3.2. MTT assay following treatment for 6 and 72 hours with [Au(tht)Cl] and [^198^Au][Au(tht)Cl], of A) MCF7 (1-20 µM, n = 2) and B) PC3 (1-100 µM, n=3) cells. Data represent percentage average cell viability (± SD) relative to controls


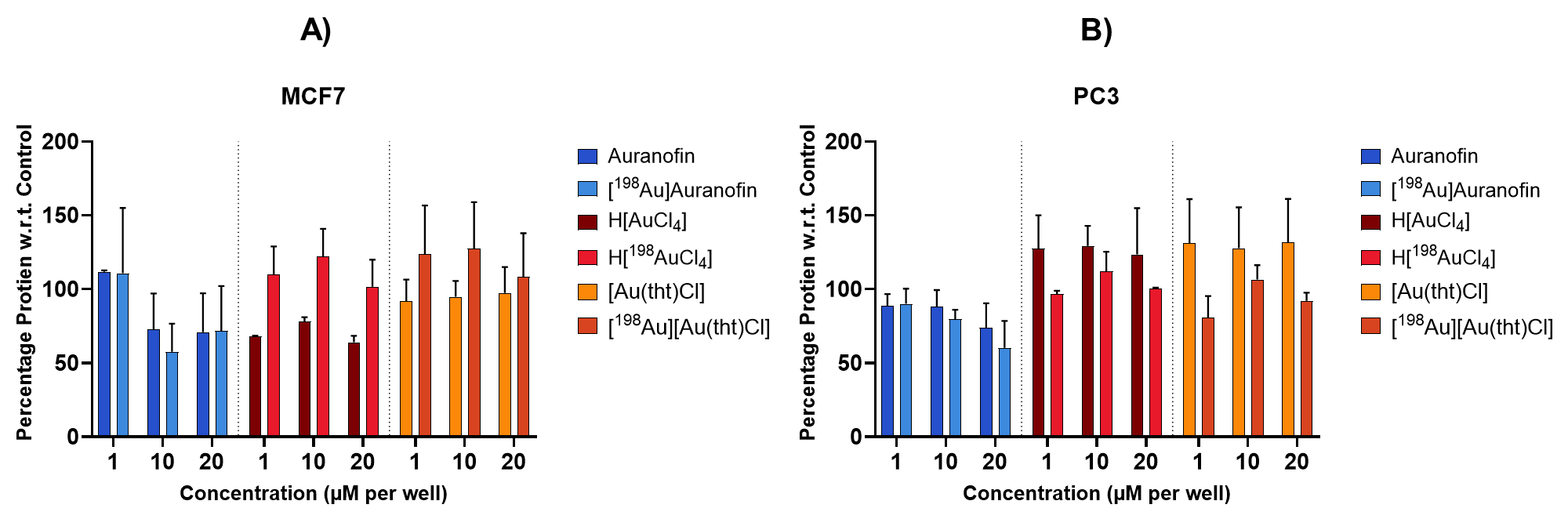


## Figure S3.3. BCA assay following treatment for 6 hours with 1 µM, 10 µM and 20 µM of radioactive and non-radioactive Auranofin, H[AuCl_4_] and [Au(tht)Cl], of A) MCF7 and B) PC3 cells. Data represent percentage average cell protein in µg/mL (± SD) relative to controls (n = 2)


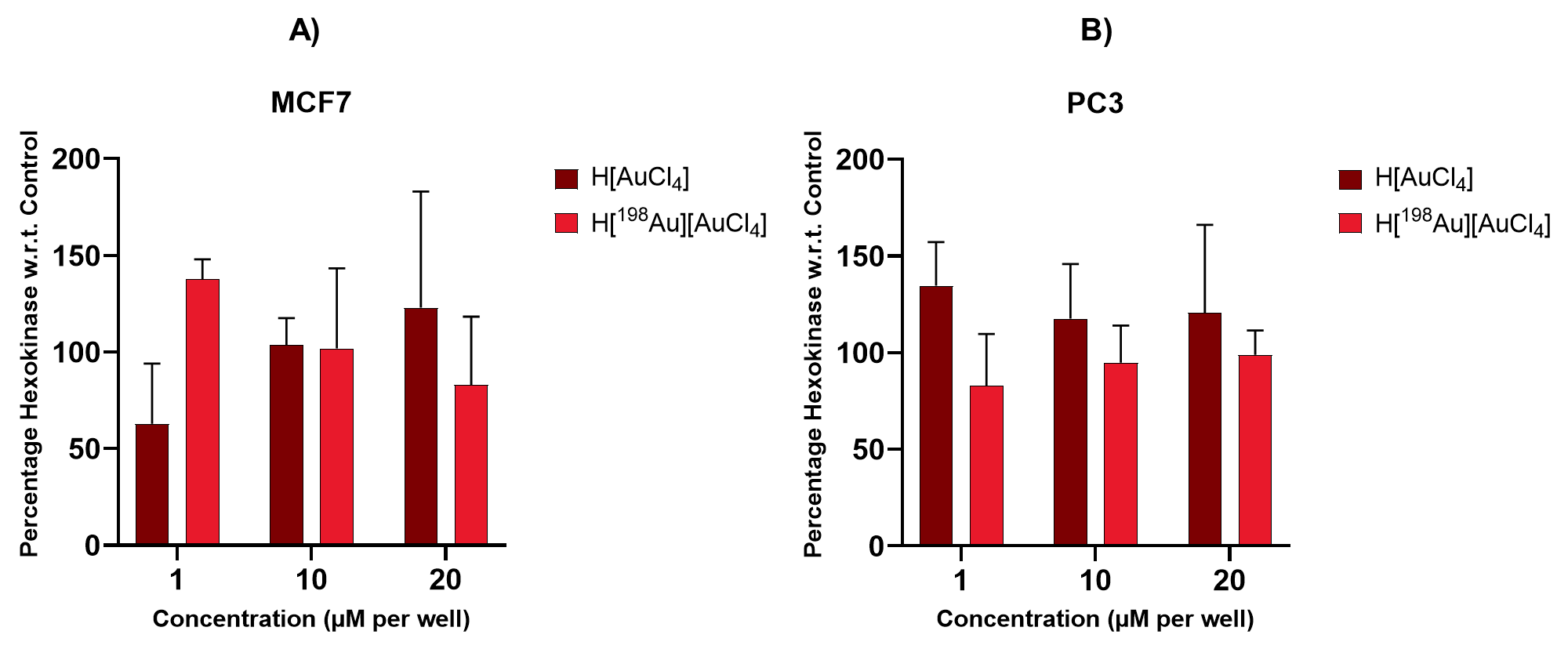


Figure S3.4. Hexokinase assay following treatment for 6 hours with 1 µM, 10 µM and 20 µM of H[AuCl4] (n = 4) and H[^198^Au][AuCl4] (n = 2) of A) MCF7 and B) PC3 cells. Data represent percentage average hexokinase activity (± SD) relative to controls


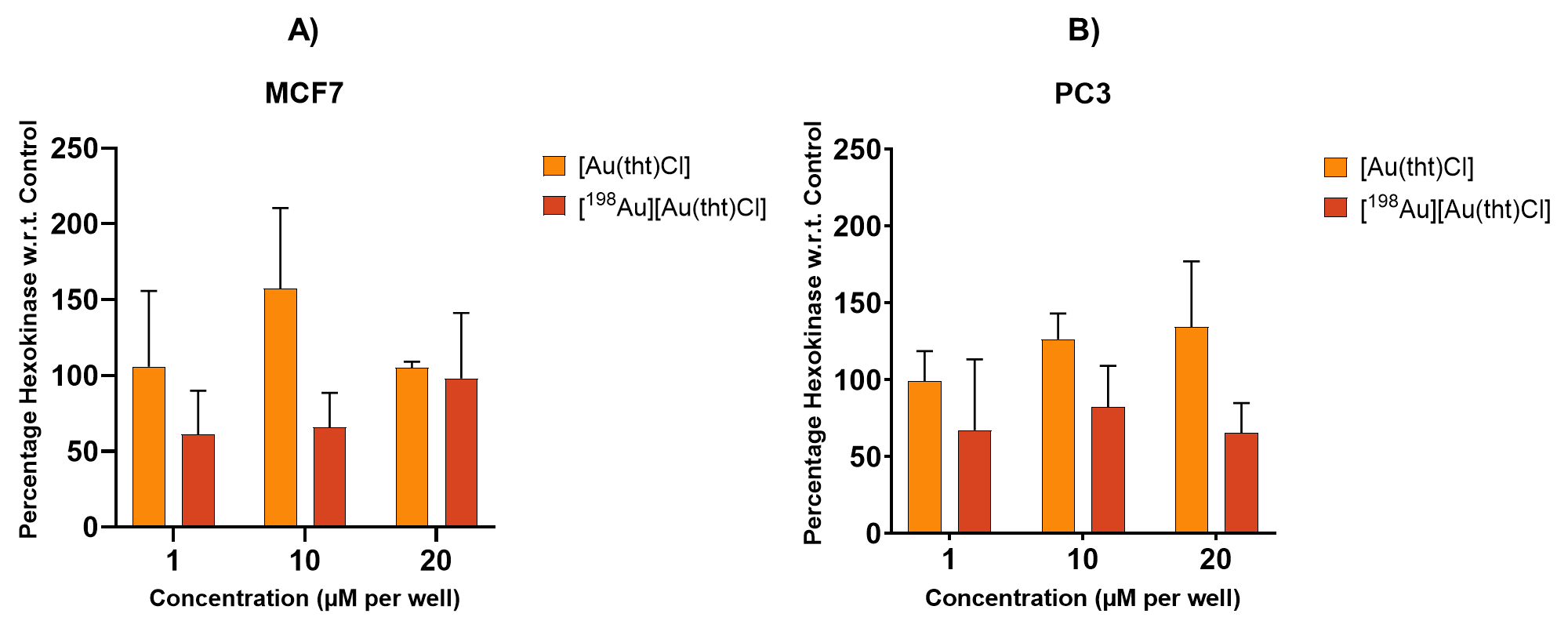


Figure S3.5. Hexokinase assay following treatment for 6 hours with 1 µM, 10 µM and 20 µM of [Au(tht)Cl] (n = 4) and [^198^Au][Au(tht)Cl] (n = 2) of A) MCF7 and B) PC3 cells. Data represent percentage average hexokinase activity (± SD) relative to controls


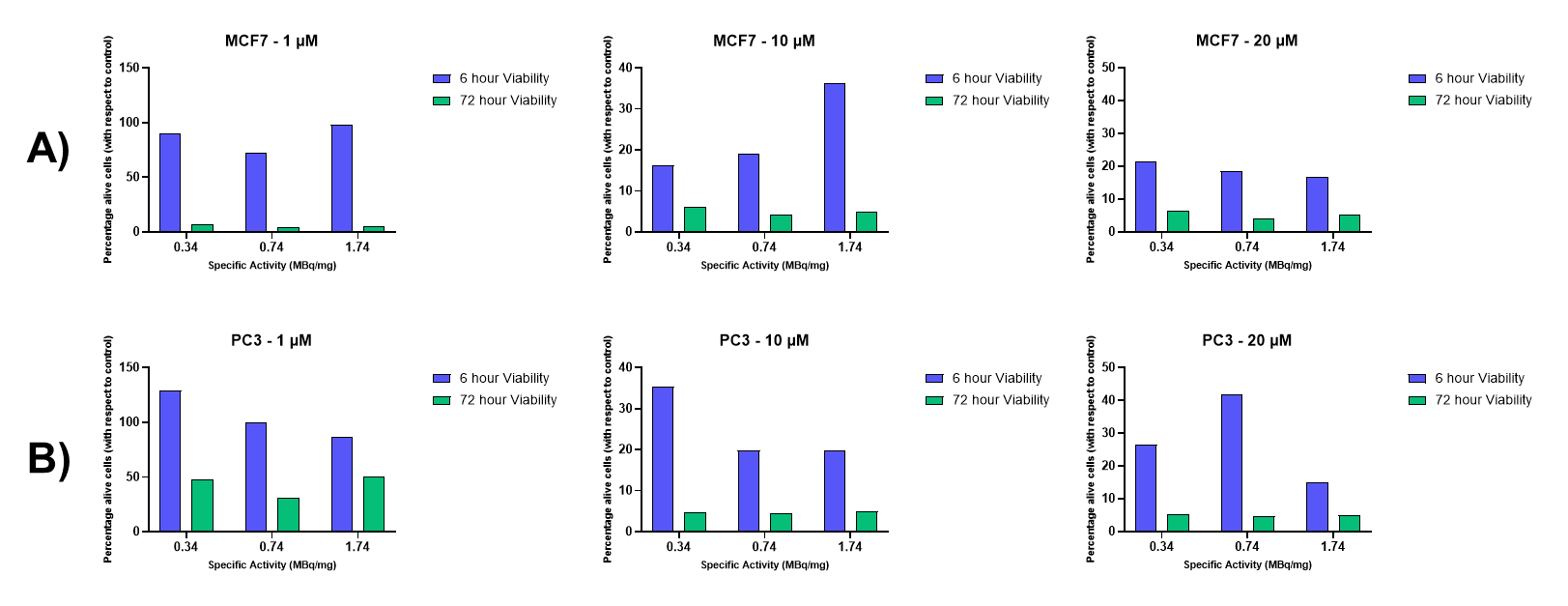


Figure S3.6. MTT assay following treatment for 6 and 72 hours with 1 µM, 10 µM and 20 µM of [¹⁹⁸Au]Auranofin plotted against the corresponding specific activity (MBq/mg) for A) MCF7 and B) PC3 cells. Data represent percentage cell viability relative to controls


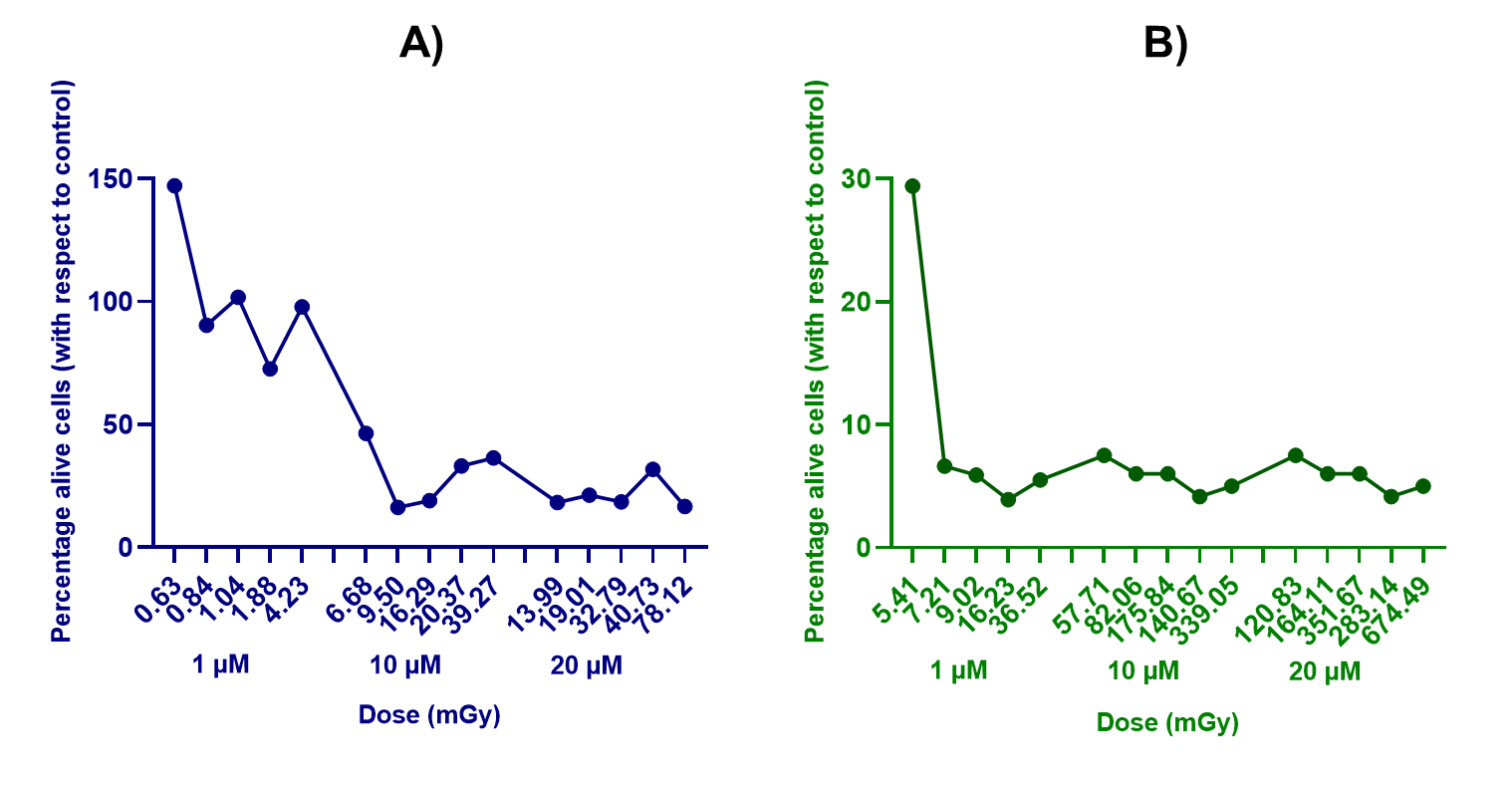


Figure S3.7. Viability results of MCF7 cells (MTT assay) after [¹⁹⁸Au]Auranofin treatment for an incubation time of A) 6 hours and B) 72 hours with 1 µM, 10 µM and 20 µM of [¹⁹⁸Au]Auranofin plotted against the corresponding dose received (mGy) per well. Data represent percentage cell viability relative to controls.
